# Quantitative susceptibility mapping identifies hippocampal and other subcortical grey matter tissue composition changes in temporal lobe epilepsy

**DOI:** 10.1002/hbm.26432

**Published:** 2023-07-26

**Authors:** Oliver C. Kiersnowski, Gavin P. Winston, Lorenzo Caciagli, Emma Biondetti, Maha Elbadri, Sarah Buck, John S. Duncan, John S. Thornton, Karin Shmueli, Sjoerd B. Vos

**Affiliations:** ^1^ Department of Medical Physics and Biomedical Engineering University College London London UK; ^2^ Department of Clinical and Experimental Epilepsy University College London London UK; ^3^ Department of Medicine, Division of Neurology Queen's University Kingston Canada; ^4^ Department of Bioengineering University of Pennsylvania Philadelphia Pennsylvania USA; ^5^ Department of Neuroscience, Imaging and Clinical Sciences Institute for Advanced Biomedical Technologies, “D'Annunzio” University of Chieti‐Pescara Chieti Italy; ^6^ Department of Neurology Queen Elizabeth Hospital Birmingham UK; ^7^ Neuroradiological Academic Unit UCL Queen Square Institute of Neurology, University College London London UK; ^8^ Centre for Microscopy, Characterisation, and Analysis The University of Western Australia Nedlands Australia; ^9^ Centre for Medical Image Computing, Computer Science department University College London London UK

**Keywords:** hippocampal sclerosis, quantitative MRI, quantitative susceptibility mapping, refractory epilepsy, temporal lobe epilepsy

## Abstract

Temporal lobe epilepsy (TLE) is associated with widespread brain alterations. Using quantitative susceptibility mapping (QSM) alongside transverse relaxation rate ($R_{2}^{*}$), we investigated regional brain susceptibility changes in 36 patients with left‐sided (LTLE) or right‐sided TLE (RTLE) secondary to hippocampal sclerosis, and 27 healthy controls (HC). We compared three susceptibility calculation methods to ensure image quality. Correlations of susceptibility and $R_{2}^{*}$ with age of epilepsy onset, frequency of focal‐to‐bilateral tonic–clonic seizures (FBTCS), and neuropsychological test scores were examined. Weak‐harmonic QSM (WH‐QSM) successfully reduced noise and removed residual background field artefacts. Significant susceptibility increases were identified in the left putamen in the RTLE group compared to the LTLE group, the right putamen and right thalamus in the RTLE group compared to HC, and a significant susceptibility decrease in the left hippocampus in LTLE versus HC. LTLE patients who underwent epilepsy surgery showed significantly lower left‐versus‐right hippocampal susceptibility. Significant $R_{2}^{*}$ changes were found between TLE and HC groups in the amygdala, putamen, thalamus, and in the hippocampus. Specifically, decreased *R*_2_* was found in the left and right hippocampus in LTLE and RTLE, respectively, compared to HC. Susceptibility and $R_{2}^{*}$ were significantly correlated with cognitive test scores in the hippocampus, globus pallidus, and thalamus. FBTCS frequency correlated positively with ipsilateral thalamic and contralateral putamen susceptibility and with $R_{2}^{*}$ in bilateral globi pallidi. Age of onset was correlated with susceptibility in the hippocampus and putamen, and with $R_{2}^{*}$ in the caudate. Susceptibility and $R_{2}^{*}$ changes observed in TLE groups suggest selective loss of low‐myelinated neurons alongside iron redistribution in the hippocampi, predominantly ipsilaterally, indicating QSM's sensitivity to local pathology. Increased susceptibility and $R_{2}^{*}$ in the thalamus and putamen suggest increased iron content and reflect disease severity.

## Graphical abstract

We used quantitative susceptibility mapping to assess, for the first time, brain tissue composition in temporal lobe epilepsy (TLE). We found significant regional susceptibility and *R*_2_* differences between control and TLE groups, and changes correlated with cognitive performance and seizure frequency.

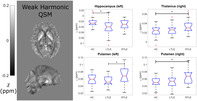

**Abbreviations**
BFR: background field removal
FAS: focal aware seizures
FBTCS: focal‐to‐bilateral tonic–clonic seizures
FIAS: Focal impaired aware seizures
GE: gradient echo
GP: globus pallidus
HC: healthy controls
HS: hippocampal sclerosis
LTLE: left temporal lobe epilepsy
QSM: quantitative susceptibility mapping
$R_{2}^{*}$
: transverse relaxation rate
ROI: region of interest
RTLE: right temporal lobe epilepsy
TLE: temporal lobe epilepsy
TV: total variation
WH‐QSM: weak harmonic quantitative susceptibility mapping

## 1 INTRODUCTION

Temporal lobe epilepsy (TLE) is the most common type of focal epilepsy. Hippocampal sclerosis (HS) is the most common histopathological cause of TLE (Prayson, 2018), and is characterized by atrophy and loss of internal tissue architecture on neuroimaging and, microscopically, by neuronal cell loss and gliosis (Özkara & Aronica, 2012). Magnetic resonance imaging (MRI) is a key tool in the diagnosis of HS, with hippocampal atrophy and signal hyperintensities on $T_{2}$‐weighted images seen in most patients (Özkara & Aronica, 2012). Further improvements in diagnostic performance have been obtained from quantification of MRI abnormalities (Goodkin et al., 2021).

Although seizures in TLE arise focally from the temporal lobe, MRI has revealed changes at cortical and subcortical levels. A recent meta‐analysis of cortical volumetry shows strong evidence for temporal and extratemporal cortical volume loss in TLE related to epilepsy disease duration (Caciagli et al., 2017), supported by a longitudinal study showing widespread cortical atrophy in TLE compared to age‐matched controls (Galovic et al., 2019). Subcortically, there are bilateral thalamic volumetric changes in TLE that relate to disease duration (Natsume et al., 2003), alterations in diffusivity properties throughout the white matter (Hatton et al., 2020), and functional connectivity changes in the thalamus and basal ganglia (Caciagli et al., 2020; He et al., 2020). Transverse relaxation rate ($R_{2}^{*}$) maps have been used to investigate the hippocampus in TLE with HS significantly associated with $R_{2}^{*}$ reductions in the hippocampus of TLE patients compared to healthy controls (HCs) but no significant $R_{2}^{*}$ differences found between TLE patients with and without HS (Santyr et al., 2017). Quantitative MRI methods such as myelin mapping and neurite density imaging have only recently seen applications in TLE, revealing widespread cortical and subcortical changes (Winston et al., 2020). Here, we explore the contributions of a different quantitative MRI technique, quantitative susceptibility mapping (QSM).

QSM (Deistung et al., 2017; Shmueli, 2020; Wang & Liu, 2015) is a quantitative MRI technique that relies on images acquired from gradient‐echo based sequences (commonly $T_{2}^{*}$‐weighted images) and calculates the tissue magnetic susceptibility distribution, χ, from the phase component, ϕ, of the complex MRI signal. There are three key steps in the QSM pipeline: (i) phase unwrapping, which removes the artificial phase wraps present in phase images due to ϕ being constrained to the $\left[-\pi ,\pi \right)$ interval; (ii) background field removal (BFR), which separates and removes the magnetic field perturbations due to external χ sources (such as the skull and air), leaving the local fields from the χ sources of interest inside the brain; (iii) susceptibility calculation from the local fields through field‐to‐source or dipole inversion. This is an ill‐posed problem solved using various mathematical regularisation strategies (Bilgic et al., 2021; Langkammer et al., 2018), each with different benefits for particular applications (Eskreis‐Winkler et al., 2017; Vinayagamani et al., 2021; Wang et al., 2017). QSM has successfully identified subtle tissue composition changes, for example, in paediatric epilepsy to reveal susceptibility changes in focal cortical dysplasia lesions, consistent with reduced iron and myelin and increased calcium and zinc content (Lorio et al., 2021). QSM has also been used to successfully derive oxygen extraction fraction maps in epilepsy patients (Ebrahimi et al., 2021), and has been suggested as a possible biomarker for diagnosis and treatment monitoring in cerebral cavernous malformations (Adamczyk et al., 2021), a common cause of epilepsy. Furthermore, QSM has been used to investigate changes in susceptibility in the presumed seizure‐onset zone between postictal and interictal states in three subjects with TLE, where increased susceptibility was found postictally compared to interictally (Zimmer et al., 2021).

Here, we extend the application of QSM in epilepsy by investigating susceptibility changes in the hippocampus, amygdala, thalamus, and basal ganglia in people with TLE and unilateral HS. Further, we compared three susceptibility calculation methods with respect to the quality of their corresponding susceptibility maps, to ensure adequate noise and residual BFR. We also included comparisons of $R_{2}^{*}$, because $R_{2}^{*}$ and susceptibility provide complementary information regarding the underlying tissue composition changes. Finally, we correlated χ and $R_{2}^{*}$ with clinical characteristics – including neuropsychology data, age of disease onset, and seizure type and frequency – to assess the potential sensitivity of these quantitative MRI measures to disease severity.

## 2 MATERIALS AND METHODS

### 2.1 Participants

We included a total of 41 participants with TLE and unilateral HS, who attended the Chalfont Centre for Epilepsy at Chalfont St Peter, Buckinghamshire, United Kingdom for routine examination. We also included 29 HCs. Visual inspection showed poor image quality due to artefacts (Supplementary Figure 1) in five TLE participants and two controls. Therefore, the final cohort consisted of 36 TLE participants and 27 HCs (see Table 1 for demographics). Nine patients underwent anterior temporal lobectomy. This project was approved by the London–Bloomsbury Research Ethics Committee (REC reference: 20/LO/0149) and comprised retrospective research conducted on clinically acquired data that did not pose risk to any patients. Written informed consent was obtained from each HC through studies approved by the National Hospital for Neurology and Neurosurgery and the UCL Institute of Neurology Joint Research Ethics Committee. For TLE participants, the following clinical characteristics were available: seizure type and frequency, disease duration, and age of epilepsy onset. For focal‐to‐bilateral tonic–clonic seizures (FBTCS) (Caciagli et al., 2020; He et al., 2020), this was further specified depending on whether patients had FBTCS in the 12 months preceding the MRI scan (called the ‘recent’ group), only longer than 12 months ago (the ‘historic’ group), or never, as in Caciagli et al. (2020).

**TABLE 1:** Demographic information for each group.

	Healthy controls (*n* = 27)	Left TLE (*n* = 19)	Right TLE (*n* = 17)
*Age*			
Range; median (IQR), years	16.5–55.1; 30 (9.6)	19.4–66.5; 32.9 (15.9)	21.4–67.1; 34.0 (16.3)
*Sex*			
Female/male, *n*	9/18	7/12	8/9
*Surgery*			
Yes/no, *n*	N/A	7/12	2/15
*Age at onset* ^a^			
Median (IQR), years	N/A	10.0 (16.5)	15.0 (16)
*Epilepsy duration* ^a^			
Median (IQR), years	N/A	25.8 (29.9)	18.0 (21.2)
*History of SE*			
Yes/no/unknown, *n*	N/A	1/8/10	2/8/7
*FAS*			
Yes/no/unknown, *n*	N/A	10/3/6	7/2/8
*FIAS*			
Yes/no/unknown, n	N/A	14/0/5	15/1/1
*FBTCS*			
Recent/historic/none	N/A	7/8/4	8/5/4
*FBTCS frequency* ^b^			
Median (IQR), per month	N/A	0.75 (0.65)	2.50 (4.0)

Abbreviations: FAS, focal aware seizures; FBTCS, focal‐to‐bilateral tonic–clonic seizures, with ‘recent’ meaning within the last 12 months, ‘historic’ means ever but not in the last 12 months; FIAS, focal impaired aware seizures; IQR, inter‐quartile range; SE, status epilepticus; TLE, temporal lobe epilepsy.

^a^ Indicates missing data (4 for left TLE, 2 for right TLE).

^b^ Indicates missing data (12 for left TLE, 9 for right TLE).

### 2.2 Data acquisition and processing

All subjects were imaged on a 3T General Electric Discovery MR750 scanner with a 32‐channel head RF receive coil. Sequences included a $T_{1}$‐weighted inversion recovery fast spoiled gradient‐recalled echo [TE/TR/TI = 3.1/7.4/400 ms, field of view (FOV) 224×256×256mm, matrix size 224×256×256, 1‐mm isotropic voxel size, parallel imaging factor = 2; acquisition time 4 min 19 s]. Subjects also underwent a multi‐echo 3D gradient‐echo (SWAN) sequence, acquired with oblique axial acquisition along the AC‐PC line, with monopolar readout gradients, in which the complex (magnitude and phase) images were saved (TE1/ΔTE/TE5 = 12.9/5.0/32.8 ms, TR = 37.1 ms, flip angle = 15°, FOV 200×200×137mm, matrix size 384×384×114, reconstructed to a voxel size of 0.52×0.52×0.60mm through zero‐padding by a factor of 2 in the last dimension; acquisition time 6 min 30 s).

**FIGURE 1:**
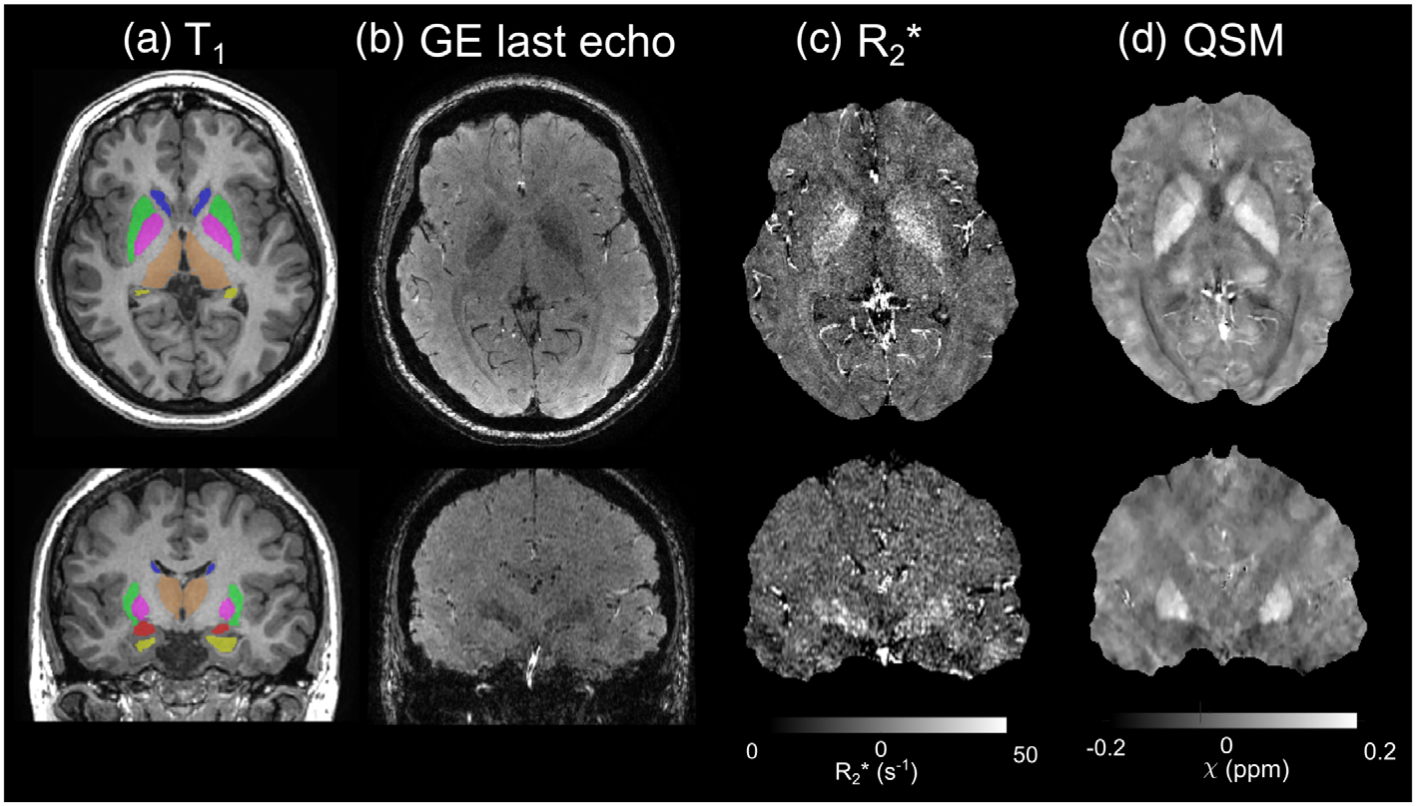
Images from a representative subject: T_1_‐weighted image, gradient echo magnitude image, *R*_2_* map and susceptibility map. (a) T_1_‐weighted image with regions of interest (ROIs) superimposed (putamen – green, globus pallidus – pink, caudate nucleus – blue, thalamus – brown, amygdala – red, hippocampus – yellow), (b) last echo gradient echo magnitude image, (c) $R_{2}^{*}$ map, (d) susceptibility (*χ*) map calculated with the optimised weak harmonic quantitative susceptibility mapping (QSM) method.

Regions of interest (ROIs) in the amygdala, caudate nucleus, globus pallidus (GP), putamen, and thalamus were segmented on the 3D $T_{1}$‐weighted images using GIF (NiftyWeb, n.d.; Cardoso et al., 2015; Prados Carrasco et al., 2016). To ensure accurate hippocampal segmentation in the presence of hippocampal pathology, HippoSeg (Winston et al., 2013) was used to segment the hippocampus. The $T_{1}$‐weighted images were rigidly registered to the first‐echo magnitude image of the QSM SWAN data using NiftyReg (Modat et al., 2014). The resulting transformation was then used to align the ROIs with the SWAN data (Figure 1).

$R_{2}^{*}$ maps were calculated via a least‐squares linear fit of the logarithm of the magnitude images over echo times using the FANSI toolbox (FANSI Toolbox, n.d.).

### 2.3 Neuropsychological testing

People with TLE underwent neuropsychological tests providing measures of: verbal comprehension (vocabulary and similarity subtests of the Wechsler Adult Intelligence Scale [WAIS]), working memory (digit span and arithmetic subtests of the WAIS), information processing (coding and matrix reasoning subtests of the WAIS), letter and category fluency, visual confrontation naming (McKenna Graded Naming Test), verbal and visual learning and recall (list and design A1‐A5 and A6 subtasks of the BIRT Memory and Information Processing Battery). A comprehensive description of these neuropsychological tests has been provided elsewhere (Ratcliffe et al., 2020).

### 2.4 Comparison of QSM methods

For all subjects, a total field map and a noise map were obtained from a non‐linear fit of the complex SWAN data over all echo times (MEDI Toolbox, n.d.; Liu et al., 2013). Residual phase wraps were removed with Laplacian unwrapping (MEDI Toolbox, n.d.; Schofield & Zhu, 2003) and a brain mask was obtained via Otsu thresholding (Otsu, 1979) on the final echo of the SWAN magnitude images. The final echo was chosen as it provides a conservative brain mask estimate, removing regions of signal dropout near areas of high susceptibility gradients. To remove other noisy regions, the brain mask was eroded via thresholding at the mean of the inverse noise map (MEDI Toolbox, n.d.; Karsa et al., 2019; Kressler et al., 2010) except within ROIs. To account for oblique slice acquisition, the total field map was rotated into alignment with the scanner axes, using FSL FLIRT (Jenkinson et al., 2012) with trilinear interpolation, after phase unwrapping and prior to BFR (Kiersnowski et al., 2022). The brain mask was then eroded by three voxels to improve the performance of BFR using projection onto dipole fields (PDF) (Liu et al., 2011).

The clinical multi‐echo SWAN data were acquired using a sequence optimised for susceptibility weighted imaging with parameters that were not appropriately optimised for QSM, which is a common issue for QSM analyses on, retrospectively, acquired clinical data. Acquired volumes had particularly high non‐isotropic, spatial resolution and suffered from low signal‐to‐noise ratio (SNR) per volume, as well as residual background fields (Schweser et al., 2017). Therefore, to reduce the impact of noise and residual background field artefacts, susceptibility maps calculated using three separate local field‐to‐susceptibility inversion methods were compared. There are a range of dipole inversion methods to choose from and, after comparison of several state‐of‐the‐art direct and iterative methods, iterative Tikhonov regularisation (Karsa et al., 2020), non‐linear total variation (TV; Milovic et al., 2018) and weak harmonic QSM (WH‐QSM) (Milovic et al., 2019) were selected. Iterative Tikhonov was chosen for its applicability to head (and neck) imaging (Karsa et al., 2020) and its use in clinical QSM research (Murdoch, Stotesbury, Kawadler et al., 2022; Murdoch, Stotesbury, Hales et al., 2022). Total variation‐based approaches were shown to be the most accurate in the QSM Challenge 2.0 (Bilgic et al., 2021) and non‐linear TV (FANSI), in particular, was chosen because it scored the highest in Stage 2 of the Challenge. WH‐QSM was also investigated due to its additional ability to remove residual background fields. Further information for each method is given below.

### 2.5 Iterative Tikhonov regularisation

The first method, iterative fitting with Tikhonov regularisation (Karsa et al., 2020), was chosen as it has shown high repeatability in head and neck images (Karsa et al., 2020). It aims to minimise the energy of susceptibility solutions by solving the minimisation problem

(1) $$\underset{\chi }{\arg\;\min}{\left\|\mathrm{MW}\left(\Delta B_{z}\left(r\right)-B_{0}\chi \left(r\right)*d_{z}\left(r\right)\right)\right\|}_{2}^{2}+\alpha {\left\|\chi \right\|}_{2}^{2},$$

where the first term is the data fidelity term reflecting the difference between the forward field calculation and the measured MRI signal, $\Delta B_{z}\left(r\right)$ is the measured local magnetic field, $B_{0}$ is the magnetic field strength, $d_{z}\left(r\right)$ is the unit magnetic dipole, $\chi \left(r\right)$ is the tissue susceptibility distribution, M is the brain mask, W (the reciprocal of the noise map) is a weighting term accounting for spatially varying noise, and α is the Tikhonov regularisation parameter. The latter was set to α=.0652 by averaging the results of an L‐curve analysis in 10 randomly selected subjects (Hansen, 1995).

### 2.6 Non‐linear TV

The second method, non‐linear TV, scored highly in the QSM Challenge 2.0 (Bilgic et al., 2021). It solves a non‐linear version of Equation (1), moving from a Gaussian noise representation to a more realistic complex‐valued Gaussian noise distribution for MRI measurements (Gudbjartsson & Patz, 1995), with TV regularisation which promotes piece‐wise constant solutions:

(2) $$\begin{array}{c} \underset{\chi }{\arg\;\min}{\left\|W\left(e^{i\left(B_{0}\chi \left(r\right)*d_{z}\left(r\right)\right)}-e^{i\Delta B_{z}\left(r\right)}\right)\right\|}_{2}^{2}+\alpha {\left|\nabla \chi \right|}_{1}. \end{array}$$

Equation (2) was solved using the FANSI toolbox (FANSI Toolbox, n.d.; Bilgic et al., 2015; Milovic et al., 2018) with the default convergence tolerance (0.1). The regularisation parameter $\alpha =1.956\times 10^{-5}$ was chosen by averaging the results of an L‐curve and frequency spectrum analysis (Milovic et al., 2021) in the same 10 subjects as for iterative Tikhonov regularisation.

### 2.7 Weak harmonic non‐linear TV

The third method, known as WH‐QSM, contains an additional regularisation term to remove residual background field artefacts (Milovic et al., 2019). This solves the minimisation problem

(3) $$\begin{array}{c} \underset{\chi ,\phi_{h}}{\arg\;\min}{\left\|W\left(e^{i\left(B_{0}\chi \left(r\right)*d_{z}\left(r\right)+\phi_{h}\left(r\right)\right)}-e^{i\Delta B_{z}\left(r\right)}\right)\right\|}_{2}^{2}+\frac{\beta }{2}{\left\|M\nabla^{2}\phi_{h}\right\|}_{2}^{2}+\alpha {\left|\nabla \chi \right|}_{1}, \end{array}$$

which is the same as Equation (2) but with an additional WH term, where $\phi_{h}$ contains residual background fields after BFR with PDF. These fields are forced to be harmonic through the WH penalty term, with β as the WH regularisation parameter, which was set to the default value (150). This value was empirically checked to ensure that only residual background fields, and no anatomical information, were contained within the harmonic field maps $\phi_{h}$. As in the non‐linear TV formulation $\alpha =1.956\times 10^{-5}$ was chosen.

### 2.8 Statistical analyses

In all analyses, *p* < .05 was used to determine statistical significance unless stated otherwise. Normality of the variables was tested using the Lilliefors goodness‐of‐fit test of composite normality, using *p* < .01 to determine statistical significance. Comparison of demographic data between study groups was performed using the Kruskal–Wallis test for continuous variables (age, age at onset, seizure frequency) and the chi‐square test for categorical variables (sex, history of status epilepticus, seizure type).

As χ and $R_{2}^{*}$ are known to depend on age (Li et al., 2014; Zhang et al., 2018) and to account for possible age differences between groups, mean χ and $R_{2}^{*}$ values in the ROIs were corrected for age. A linear age correction was chosen as there was large variability in ROI mean values and the quality of the linear fit was far greater than using an exponential model. Age‐correction used a least‐squares linear fit across control participants in each ROI (Acosta‐Cabronero et al., 2016), pooled across both hemispheres

(4) $$\begin{array}{c} \hat{Y_{i}}=\lambda_{i}+\theta_{i}A \end{array}$$

where $\hat{Y_{i}}$ is the mean value (χ or $R_{2}^{*}$) in an ROI i, A is the age, and $\lambda_{i}$ and $\theta_{i}$ are the fitted parameters. The age‐corrected mean value in each ROI i and TLE subject j, $\chi_{i,j}$, is given by:

(5) $$\begin{array}{c} \chi_{i,j}=Y_{i,j}+\theta_{i}\left(\mu -A_{j}\right) \end{array}$$

where $Y_{i,j}$ is the measured mean value, μ is the mean age in the control group, and $A_{j}$ is the age of subject j.

### 2.9 QSM quality

To quantitatively compare the noise levels within ROIs of susceptibility maps calculated using the three different inversion methods, the standard deviation of χ was calculated in each ROI of each subject and a three‐group two‐tailed ANOVA was performed to compare the average standard deviation between the three susceptibility calculation techniques: iterative Tikhonov regularisation, non‐linear TV and WH‐QSM.

### 2.10 Comparing ROI mean χ and $R_{2}^{*}$ between groups and hemispheres

Three‐group two‐tailed ANOVA was then performed for each ROI, testing for significant differences in χ and $R_{2}^{*}$ values between the LTLE, RTLE, and control groups, using *η* ^2^ to denote the effect size. Post hoc Tukey–Kramer tests were used to assess which groups exhibited statistically significant differences if ANOVA revealed group differences, which incorporates multiple comparison correction. Here, Cohen's *d* is used to denote the effect size. Additionally, intra‐subject left–right differences in ROI mean values were investigated per group using a paired t test, using Cohen's *d* to denote the effect size.

To ensure any regional differences found were not driven by age, individual linear fits of ${\chi /R}_{2}^{*}$ versus age for the three groups were compared using analysis of covariance in all ROIs (pooled across both hemispheres).

### 2.11 Correlation with clinical features

In TLE, age of onset is correlated with various MRI‐based biomarkers (e.g., cortical thinning (Galovic et al., 2019)) so we explored correlations between ROI mean susceptibility and $R_{2}^{*}$ and age of onset. Age of epilepsy onset was distributed highly non‐normally, and as log‐transformation did not improve this we used Spearman rank correlations to investigate correlations with susceptibility or $R_{2}^{*}$.

Previous work in TLE indicates that the thalamus and basal ganglia may facilitate FBTCS (Caciagli et al., 2020; He et al., 2020). Therefore, we compared ROI mean susceptibility and $R_{2}^{*}$ across patient groups stratified based on FBTCS (none, historic, or recent) using ANOVA. In those patients with recent FBTCS we also correlated these quantitative measures with frequency of FBTCS in the year preceding the scan (Caciagli et al., 2020) using Pearson correlation. As only seven LTLE and eight RTLE patients reported recent FBTCS, data from the two patient groups were pooled by ipsilateral and contralateral ROIs, as the impact of FBTCS is considered as most prominent in the ipsilateral hemisphere (Caciagli et al., 2020; He et al., 2020).

Neuropsychological test scores were correlated with the ROI mean susceptibility and $R_{2}^{*}$ using multiple linear regressions to include covariation with patient group (LTLE vs RTLE). For some cognitive test scores, it is known that LTLE and RTLE are affected differently (O'Muircheartaigh et al., 2012; Xiao et al., 2018), therefore, we included an interaction term between patient group and cognitive test. Some cognitive processes (e.g., naming) rely on lateralized hemispheric processing, so left‐sided (LTLE) and right‐sided (RTLE) ROIs were considered in separate regressions. These regressions can reveal if imaging metrics correlate with cognitive scores (slope of regression), if the two groups (LTLE and RTLE) have a difference in average susceptibility/*R*_2_* (group effect), or a different sign/magnitude of effect between the groups (group × cognitive score interaction). As not all participants performed all tests, the number of participants included in each correlation analysis is given with the statistical test outcomes. Based on prior work highlighting the relevance of the thalamus and basal ganglia for linguistic and executive processing (O'Muircheartaigh et al., 2012; Viñas‐Guasch & Wu, 2017; Xiao et al., 2018), executive function tests (working memory, arithmetic, picture naming, and letter and category fluency) as well as information processing and verbal comprehension scores were included in a multiple regression model with the ROI mean susceptibilities or $R_{2}^{*}$ values within the caudate nucleus, hippocampus, GP, putamen, and thalamus. Finally, verbal and visual memory scores were regressed against mean hippocampal susceptibility and $R_{2}^{*}$ values. These regressions were deemed significant at *p* < .05 using the false discovery rate to correct for multiple comparisons.

In each group, we performed correlations between hippocampal volume – a known radiological biomarker of HS – and mean hippocampal χ values and between hippocampal volume and mean $R_{2}^{*}$ values to investigate if these susceptibility‐based metrics provide overlapping or new information.

## 3 RESULTS

### 3.1 Demographic and clinical characteristics

There was a significant difference in age between the three groups (Table 1; $\chi^{2}=7.96;\mathrm{df}=2;$ p=.019, $\eta^{2}=0.12$), and susceptibility values were age‐corrected as detailed above. Sex was not different between the three groups. None of the other patient characteristics were significantly different between left and right TLE groups. All surgical specimens were HS type 1.

### 3.2 QSM quality

**FIGURE 2:**
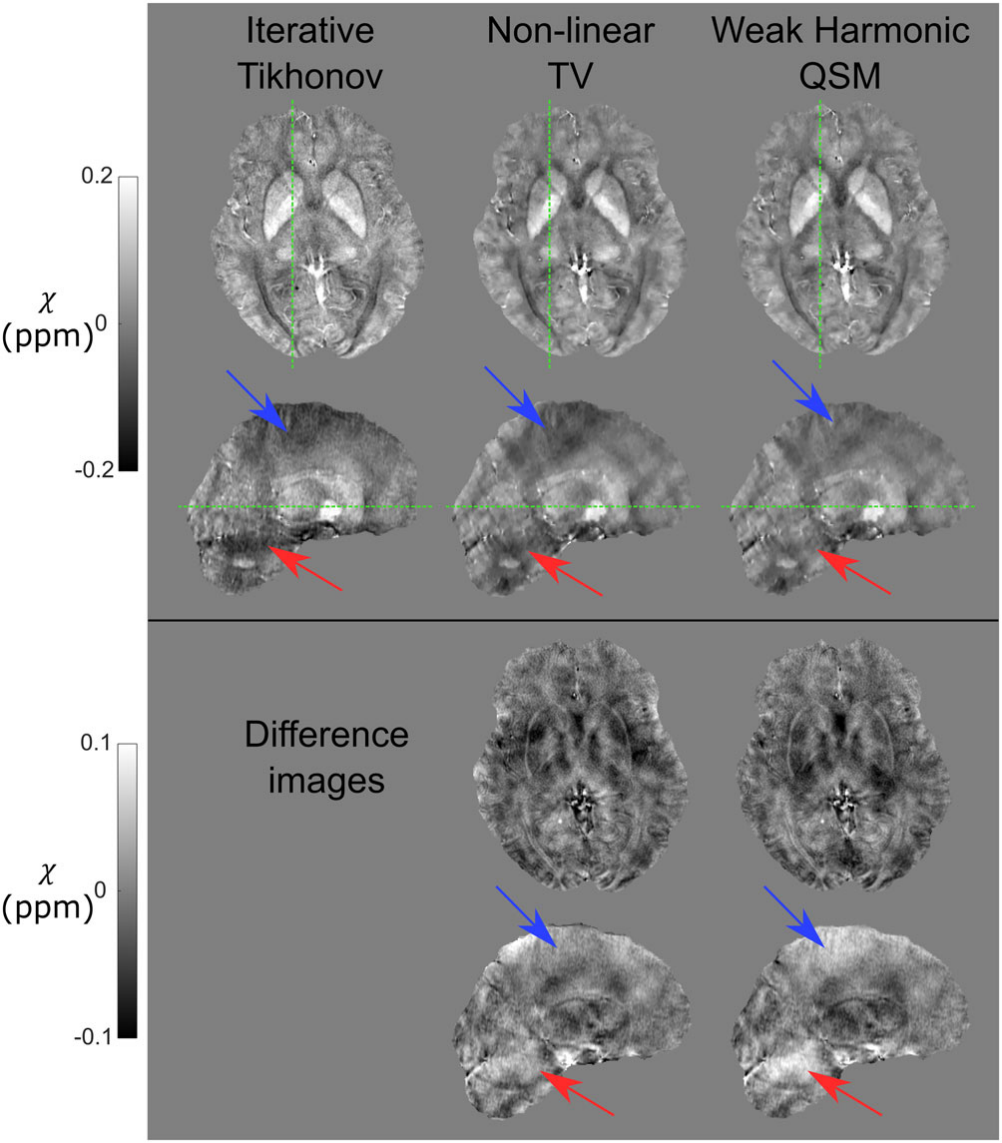
Comparison of susceptibility calculation techniques. Comparison of the three susceptibility (*χ*) calculation methods in a representative right‐sided temporal lobe epilepsy (RTLE) subject: iterative Tikhonov regularisation (left), non‐linear total variation (middle) and weak harmonic quantitative susceptibility mapping (QSM) (right). Difference images are relative to the iterative Tikhonov regularisation susceptibility map. Iterative Tikhonov suffers from high noise and residual background fields. Weak‐harmonic (WH)‐QSM performs the best, removing both noise and residual background fields. This is most evident in the cerebellum (red arrows) and the top of the brain (blue arrows). Axial and sagittal slice positions are indicated by the green dashed lines.

Susceptibility maps calculated via iterative Tikhonov regularisation suffered from noise and residual background fields, particularly in the cerebellum and the top of the brain (Figure 2). Upon visual comparison, non‐linear TV reduced noise and increased the contrast in deep grey matter ROIs (Figure 2). Residual background fields remained in the non‐linear TV susceptibility maps, and WH‐QSM qualitatively reduced the noise, reduced the standard deviation of susceptibility values within ROIs, and removed residual background fields (Figure 2).

**FIGURE 3:**
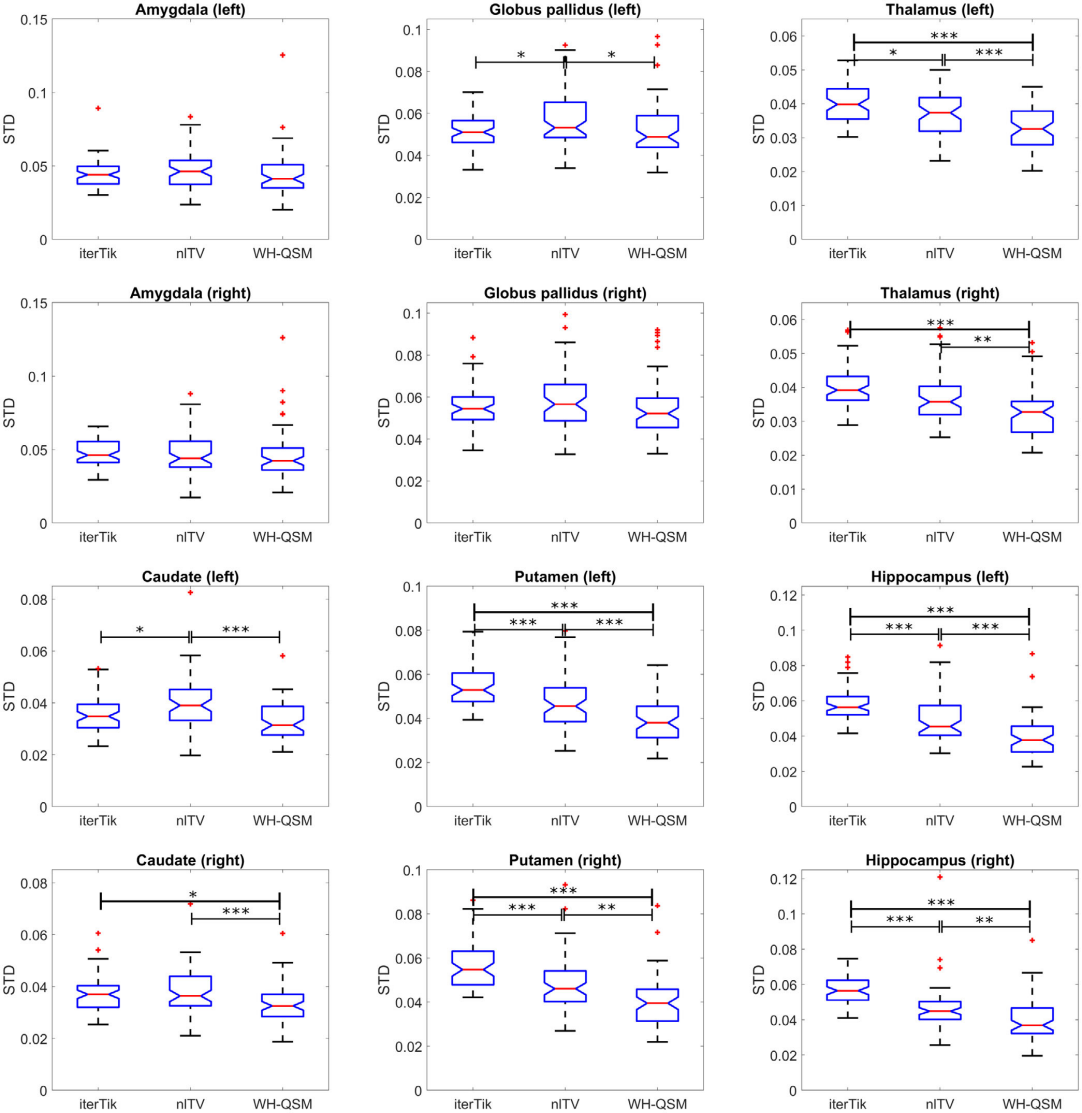
Comparison of standard deviation across susceptibility calculation techniques. The average standard deviation of susceptibility values in each region of interest (ROI), over all participants regardless of disease state, was compared across the three quantitative susceptibility mapping (QSM) methods: iterative Tikhonov regularisation (iterTik), non‐linear total variation (nlTV), and weak harmonic QSM (WH‐QSM). WH‐QSM consistently had the lowest standard deviation for all ROIs. An outlier (STD >0.25) in the left and right amygdala in the non‐linear TV group has been omitted to facilitate comparison. * indicates p<.05, ** indicates p<.01, *** indicates p<.001.

Three‐group one‐way ANOVA indicated significant standard deviation differences between the three susceptibility calculation methods in the bilateral caudate nucleus (p<.001 for both), putamen (p<.001 for both), thalamus (p<.001 for both), hippocampus (p<.001 for both), and left GP (p=.021). Tukey–Kramer multiple comparison analysis tests revealed that non‐linear TV had significantly greater standard deviation in several ROIs (Figure 3) compared to both iterative Tikhonov and WH‐QSM. It also revealed that WH‐QSM outperformed both non‐linear TV and iterative Tikhonov in all of ROIs that displayed significant differences across methods except for the left GP, where it only outperformed non‐linear TV (Figure 3).

This quality comparison identified WH‐QSM as the optimal method for these data and all group results and correlations shown are from the susceptibility maps calculated with WH‐QSM.

### 3.3 Group differences in susceptibility

**FIGURE 4:**
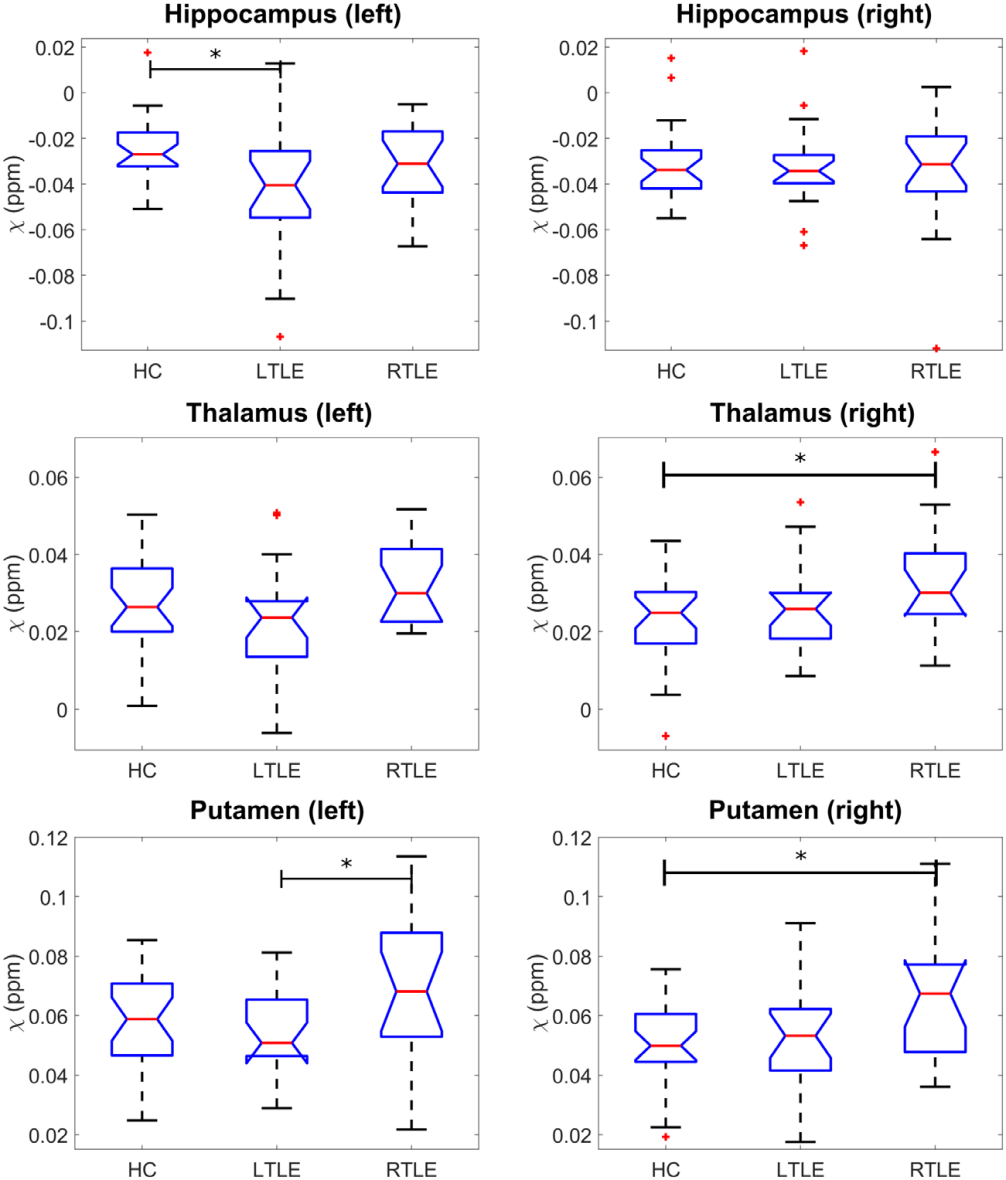
Significant region of interest (ROI) mean susceptibility differences between temporal lobe epilepsy (TLE) and healthy control groups. Boxplots showing comparison of average susceptibility (*χ*) across the three groups. * indicates p<.05. HC, healthy controls; LTLE, left temporal lobe epilepsy; RTLE, right temporal lobe epilepsy.

The susceptibility values in all ROIs in all groups were found to be normally distributed. We observed significant susceptibility differences between groups in the left hippocampus (p=.020), right thalamus (p=.049), left putamen (p=.036) and the right putamen (p=.017) using ANOVA (Figure 4, Supplementary Table 1). Tukey–Kramer multiple comparison analysis tests revealed that: the LTLE group had a significantly lower susceptibility in the left hippocampus compared to HCs (p=.015), but the RTLE group did not (p=.513). The RTLE group had a significantly higher susceptibility in the right thalamus than HCs (p=.040), but the LTLE group did not (p=.757). The RTLE group had a significantly higher susceptibility in the left putamen compared to the LTLE group (p=.041), and in the right putamen compared to HCs (p=.014). The LTLE group was not significantly different in susceptibility of the left putamen (p=.859) or the right putamen (p=.789) compared to HCs. Effect sizes and details can be found in Table 2.

**TABLE 2:** Significant results of ANOVA for group‐wise χ and $R_{2}^{*}$ comparisons. ANOVA *p*‐values, post hoc T–K *p*‐values and their corresponding effect sizes (*η* ^2^ and Cohen's *d*, respectively) for group‐wise χ and $R_{2}^{*}$ comparisons, which showed significant ANOVA differences. Bold table entries signify statistically significant differences (with post hoc T–K *p* < .05).

Susceptibility (χ)			HC vs. LTLE		HC vs. RTLE		LTLE vs. RTLE	
ROI	ANOVA	$\eta^{2}$	T–K *p*‐value	Cohen's *d*	T–K *p*‐value	Cohen's *d*	T–K *p*‐value	Cohen's *d*
Hippocampus (left)	0.020	0.122	**.015**	**0.837**	.513	0.418	.269	0.433
Putamen (left)	0.036	0.105	.859	0.186	.084	−0.623	**.041**	**0.726**
Putamen (right)	0.017	0.127	.789	−0.212	**.014**	**−0.893**	.098	0.614
Thalamus (right)	0.049	0.096	.757	−0.222	**.040**	**−0.742**	.221	0.527
$R_{2}^{*}$								
Amygdala (left)	0.003	0.177	**.004**	**1.054**	**.031**	**0.854**	.838	0.158
Amygdala (right)	0.006	0.155	.262	0.539	**.004**	**1.011**	.232	−0.465
Hippocampus (left)	0.001	0.202	**.001**	**1.207**	.904	0.136	**.013**	**0.831**
Hippocampus (right)	0.0001	0.256	.347	0.573	**.0001**	**1.289**	**.013**	**−0.797**
Putamen (left)	0.008	0.149	.195	0.584	.177	−0.514	**.005**	**1.004**
Thalamus (left)	0.007	0.153	.527	0.308	**.049**	**−0.839**	**.006**	**0.958**

Abbreviations: HC, healthy controls; LTLE, left temporal lobe epilepsy; ROI, region of interest; RTLE, right temporal lobe epilepsy; T–K, Tukey–Kramer.

We also identified left–right asymmetry in susceptibility in the putamen in the HC group, with the left putamen having a higher susceptibility than the right (p=.032) using a paired *t*‐test. No asymmetry in putamen susceptibility was observed in the LTLE or RTLE groups. Although no left–right asymmetry in susceptibility was found in the hippocampi in any of the groups, subgroup analysis within the surgical LTLE group did reveal a significantly lower susceptibility in the left (affected) hippocampus than the right (−0.050 ppm vs. −0.035 ppm, respectively, p=.031).

No statistically significant differences between groups in the analysis of covariance of susceptibility with age were found in any of the ROIs (Supplementary Figure 3), indicating that the regional differences found were not driven by age.

### 3.4 Group differences in $R_{2}^{*}$

**FIGURE 5:**
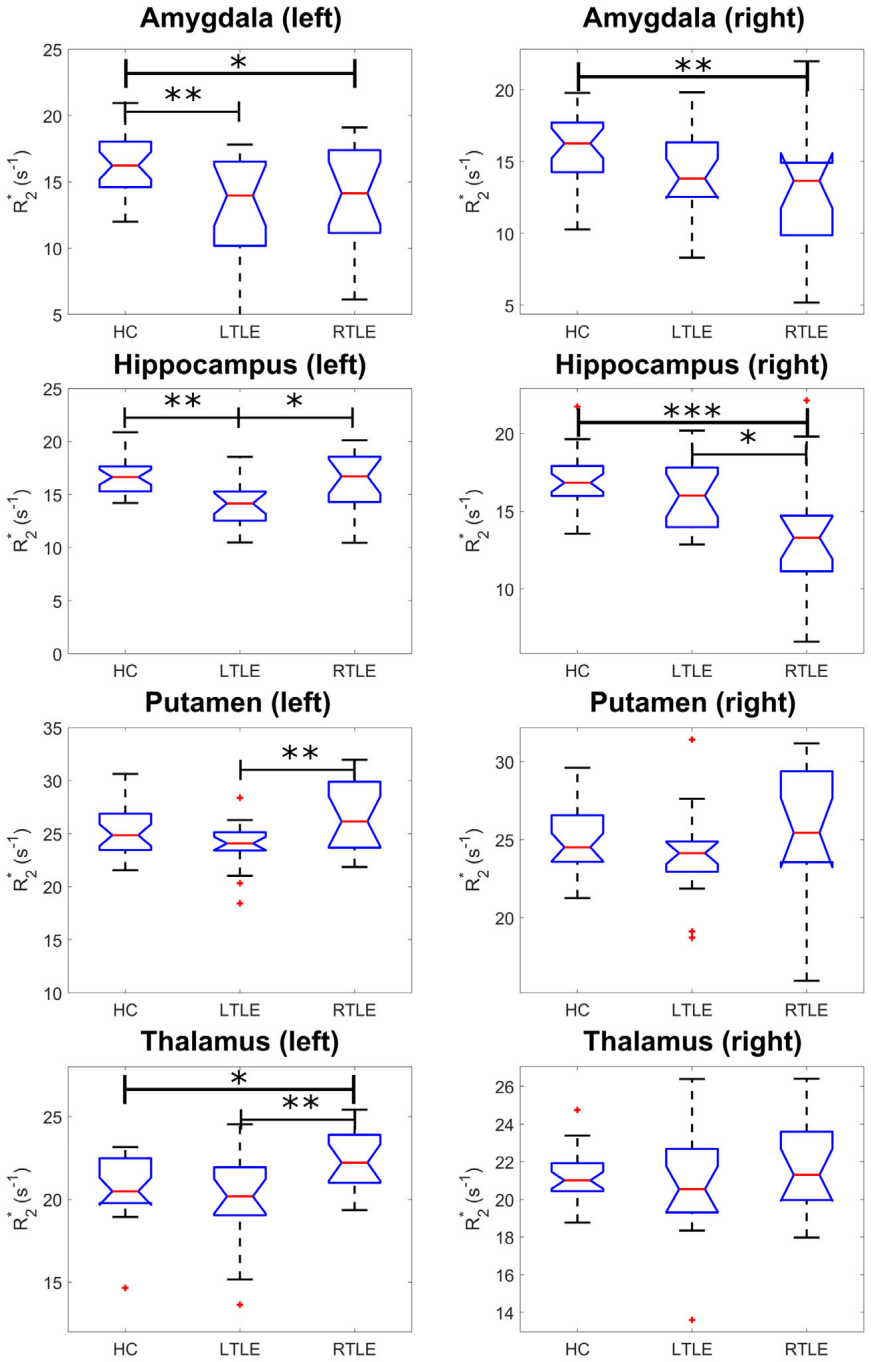
Significant region of interest (ROI) mean $R_{2}^{*}$ differences between temporal lobe epilepsy (TLE) and healthy control groups. Significant $R_{2}^{*}$ group changes in six ROIs are shown. Both pathological hippocampi in their respective TLE group were found to have significantly reduced $R_{2}^{*}$ values. * indicates p<.05, ** indicates p<.01, *** indicates p<.001.

The $R_{2}^{*}$ values in all ROIs were found to be normally distributed. With ANOVA, we observed group $R_{2}^{*}$ differences (Figure 5, Supplementary Table 1) in the left and right amygdala (p=.0029 and p=.0063, respectively), hippocampus (p=.0012 and p<.001, respectively), the left putamen (p=.0078), and the left thalamus (p=.0069).

Tukey–Kramer multiple comparison analysis tests revealed that both the LTLE and RTLE group had significantly lower $R_{2}^{*}$ in the left amygdala compared to HCs (p=.004,p=.031, respectively). The TLE groups had significantly reduced $R_{2}^{*}$ in their ipsilateral hippocampus compared to both HCs and the contralateral hippocampus (Figure 5). The left putamen was found to have a significantly higher $R_{2}^{*}$ in the RTLE group compared to the LTLE group (p=.005) but not the control group. The left thalamus had a significantly higher $R_{2}^{*}$ in the RTLE group than both the control and LTLE groups (p=.049,p=.006). Effect sizes and details can be found in Table 2.

Using paired *t* tests, we also identified left–right asymmetry in the hippocampus of the LTLE and RTLE groups, with the ipsilateral hippocampus having a lower $R_{2}^{*}$ than the contralateral hippocampus in each group (p=.0215 and p=.0265, respectively). We also identified asymmetry in the GP of the RTLE group with the left GP having a higher $R_{2}^{*}$ than the right (p=.0167).

No statistically significant differences between groups in the analysis of covariance of $R_{2}^{*}$ and age were found in any of the ROIs (Supplementary Figure 4), indicating that the regional differences found were not driven by age.

### 3.5 Correlations with clinical features

**FIGURE 6:**
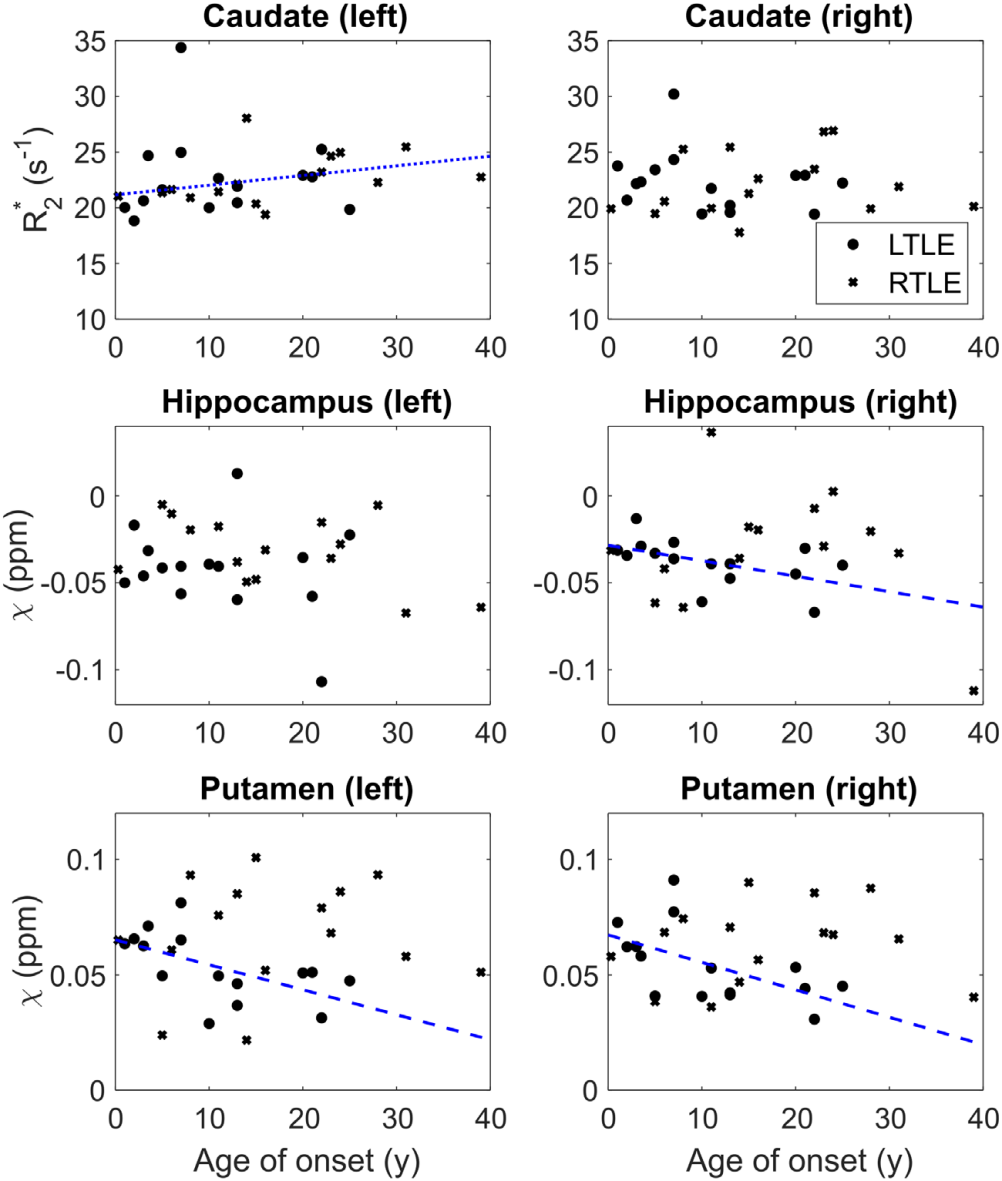
Susceptibility and $R_{2}^{*}$ versus age of temporal lobe epilepsy (TLE) onset. Scatterplots showing caudate $R_{2}^{*}$ and hippocampal and putamen susceptibility (*χ*) versus age of TLE onset. Dots indicate people with left TLE; crosses indicate people with right TLE. Dashed (left TLE) and dotted (right TLE) lines indicate plots of linear correlation for regions with significant correlations. These lines are shown only as a visual aid as significance testing was performed using Spearman rank correlation.

We found negative correlations between age of TLE onset and: bilateral putamen susceptibility (p=.013 for left; p=.028 for right) and right hippocampal susceptibility (p=.014) in the LTLE group. A positive correlation was found between the age of TLE onset and left caudate $R_{2}^{*}$ (p=.034) in the RTLE group (Figure 6).

**FIGURE 7:**
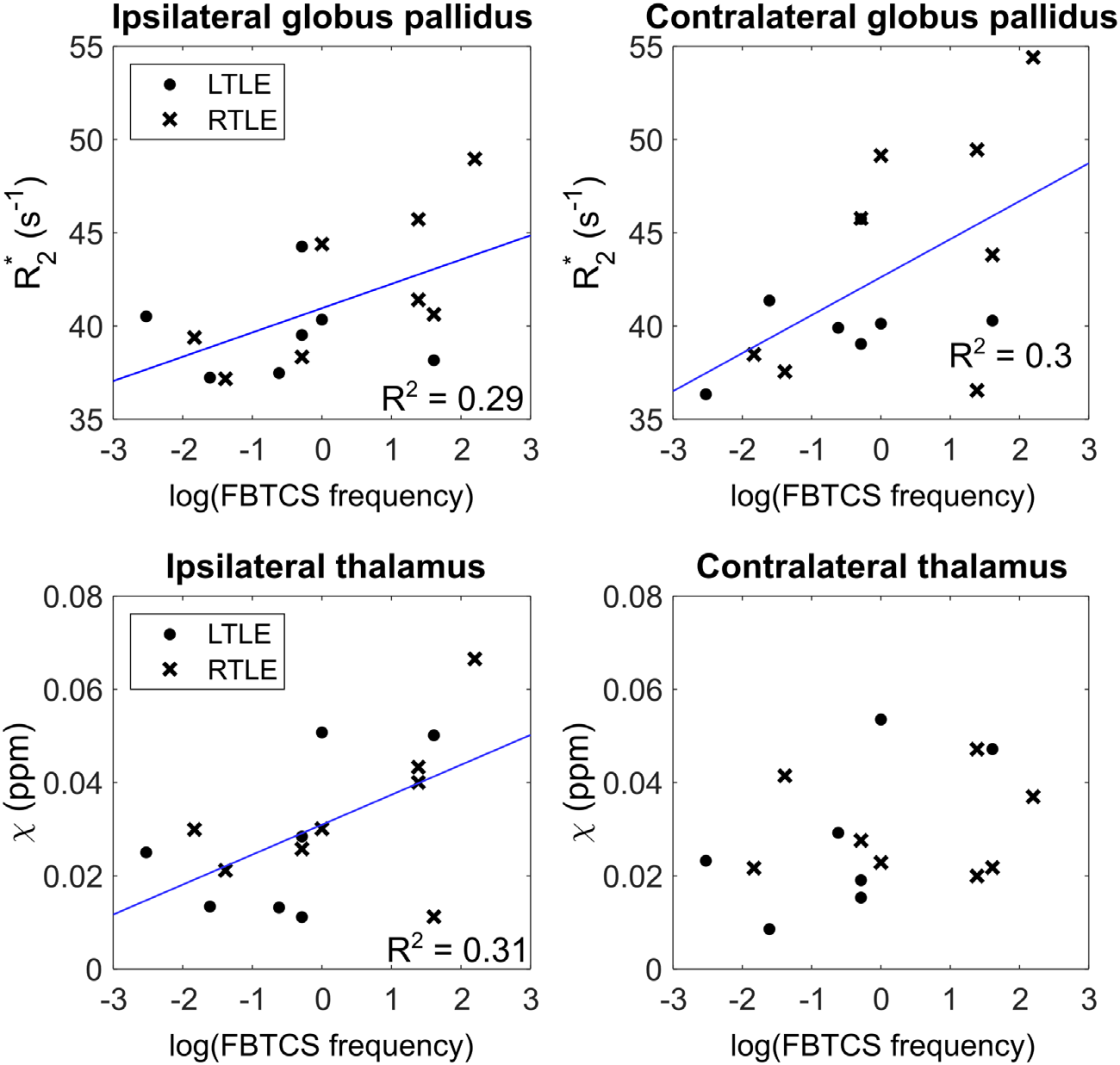
Susceptibility and $R_{2}^{*}$ versus log‐transformed frequency of focal‐to‐bilateral tonic–clonic seizures (FBTCS). Scatterplots showing thalamic and putamen susceptibility (*χ*) and $R_{2}^{*}$ in the globi pallidi against the frequency of FBTCS, log‐transformed to ensure normality. Dots indicate people with left temporal lobe epilepsy (TLE); crosses indicate people with right TLE; the black line indicates the linear fit of significantly correlated factors.

There were no significant differences in susceptibility or $R_{2}^{*}$ between FBTCS groups (recent, historic, or none). FBTCS frequency was highly non‐normal (p<.001), and data were log‐transformed to ensure normality (p=.25 after log‐transformation) and facilitate linear correlations with *χ* and $R_{2}^{*}$. There were significant positive correlations between FBTCS frequency and susceptibility in the ipsilateral thalamus (p=.031) and the contralateral putamen (p=.042), and significant positive correlations between FBTCS frequency and $R_{2}^{*}$ in the ipsilateral and contralateral globi pallidi (p=.040 and p=.036, respectively; Figure 7).

None of the neurocognitive test scores were significantly correlated with age for either patient population. Significant multiple linear regressions between neuropsychological tests and susceptibility or $R_{2}^{*}$ are summarised in Supplementary Table 2. Neuropsychological scores were normally distributed for all tests. No significant findings were observed for the right‐sided ROIs. Arithmetic performance (*n* = 18) was associated with higher left caudate susceptibility (p=.0032) and higher left putamen susceptibility (p<.001). In both regions, the RTLE patients had higher susceptibility values, a difference that diminished with higher test scores (negative interaction). Letter fluency (*n* = 32) was associated with higher left hippocampal $R_{2}^{*}$ (p=.0022) and higher left thalamic $R_{2}^{*}$ (p=.0056). For the hippocampus, RTLE patients had lower $R_{2}^{*}$ values but a stronger increase in $R_{2}^{*}$ with increasing test scores (positive interaction). For the thalamus, RTLE patients had a higher $R_{2}^{*}$ that increased further with increasing test scores (a positive interaction term). Matrix reasoning (*n* = 31) was positively associated with left thalamic $R_{2}^{*}$ (p=.029). Here, the RTLE group had higher $R_{2}^{*}$ values, but with a smaller positive association between test scores and $R_{2}^{*}$ (a negative interaction). No correlations were observed for other regions or other cognitive test scores.

Investigations of correlations between hippocampal susceptibility or $R_{2}^{*}$ and volume found a significant positive (p=.036) association between hippocampal volume and $R_{2}^{*}$ in the right hippocampus in the RTLE group (Supplementary Figure 2).

## 4 DISCUSSION

In this study, we investigated the use of QSM in a cohort of people with TLE and HCs. We revealed, for the first time, that there are in vivo susceptibility and $R_{2}^{*}$ differences between people with TLE and HCs in the amygdala, hippocampus, thalamus, and basal ganglia. We also identified correlations between susceptibility and $R_{2}^{*}$ measures and clinical characteristics (age at epilepsy onset, FBTCS frequency in the last year, and neuropsychological test scores), indicative of these quantitative MRI metrics' sensitivity to changes in tissue composition underlying disease characteristics and cognitive performance.

To provide a biological interpretation of the observed *χ* and $R_{2}^{*}$ changes, it is important to consider what these measures reflect. *χ* estimates reflect the magnetic susceptibility which, in biological tissues, is primarily influenced by myelin and iron content (Duyn & Schenck, 2017); myelin is diamagnetic (*χ* < 0, meaning that myelin reduces the local magnetic field strength), while iron is paramagnetic (*χ* > 0, meaning iron enhances the local magnetic field). However, when *χ* increases are observed, these could be the result of myelin reduction or iron accumulation. $R_{2}^{*}$ complements *χ* measurements, as $R_{2}^{*}$ is a measure of the concentration of microscopic susceptibility sources, with $R_{2}^{*}$ increases indicating an increase in susceptibility sources (e.g., increased tissue iron). This means that an observed increase in both *χ* and $R_{2}^{*}$ in a particular brain region is most parsimoniously explained by an increase in paramagnetic iron content in that region, whereas an increase in *χ* coupled with a reduction in $R_{2}^{*}$ are more indicative of reduction in diamagnetic myelin content. Other factors may contribute to *χ* and $R_{2}^{*}$ (e.g., zinc, calcium), and therefore, without histological confirmations, the above interpretations may still reflect a simplification of the true biological complexity.

### 4.1 Regional differences between groups

In people with LTLE, the left hippocampus had a significantly lower susceptibility than in the controls, and a lower mean $R_{2}^{*}$ than the right hippocampus. In the RTLE group, $R_{2}^{*}$ values were significantly lower in the right hippocampus than the left in agreement with literature (Santyr et al., 2017). Group differences may be explained by the observed within‐subject asymmetry, with both patient groups demonstrating lower ipsilateral than contralateral $R_{2}^{*}$. Further, there is a consistently more negative ipsilateral hippocampal susceptibility in the subgroup of LTLE patients who underwent surgery. Right hippocampal volume was positively correlated with right hippocampal $R_{2}^{*}$, indicating that, with more atrophy, there was a reduction in $R_{2}^{*}$ and thus a loss of susceptibility sources (e.g., myelin or iron). Hippocampal susceptibility differences were not found in the RTLE group, possibly due to a slightly smaller sample and higher within‐group standard deviation (0.0313 ppm vs. 0.0265 ppm).

Although a loss of hippocampal iron might be postulated as the simplest cause of these susceptibility and $R_{2}^{*}$ decreases, this may at first glance be complex to reconcile with the underlying biology, given that the two main histopathological hallmarks of HS are neuronal cell loss and gliosis (Thom, 2014). Neuronal cell loss, especially in HS type 1 as observed in our participants, predominantly affects CA1 and spares the subiculum (Thom, 2014); the latter is heavily myelinated, especially compared to CA1 (DeKraker et al., 2018; Krogsrud et al., 2014). Hence, we hypothesize that a loss of relatively low‐myelinated neurons in CA1 may increase the average myelination (i.e., the concentration of diamagnetic myelin) throughout the hippocampus and thus explain the decreased susceptibility values in the affected left hippocampi.

Significant decreases in $R_{2}^{*}$ in the right hippocampus of the RTLE group and the correlation between $R_{2}^{*}$ and hippocampal volume in the same RTLE group point towards demyelination accompanied by neuronal cell loss – because $R_{2}^{*}$ decreases when tissue susceptibility sources (whether diamagnetic or paramagnetic) are lost. The left hippocampus in the LTLE group also shows a decrease in $R_{2}^{*}$, indicating the same mechanism, although the latter may be confounded by the significant susceptibility decrease in the pathological hippocampus of the LTLE group. Normally, we would expect demyelination (loss of diamagnetic myelin) to be observed as an increase in susceptibility, and highly myelinated regions with negative susceptibility to thus become less negative. However, as we explained above, the decrease in hippocampal susceptibility in the LTLE group together with a decreased $R_{2}^{*}$ could be explained by a loss of low‐myelinated CA1 neurons that leaves behind the subiculum's highly myelinated neurons.

Previous studies in multiple sclerosis (Schweser et al., 2021) have also suggested that $R_{2}^{*}$ and susceptibility changes can be explained by selective loss of particular cells (e.g., iron‐rich vs. iron‐free cells or, in this study, low‐myelin versus high‐myelin neurons). Decreases in hippocampal susceptibility were also observed in premanifest Huntington Disease patients, and attributed to a possible redistribution of brain iron in response to the loss of myelin (Van Bergen et al., 2016). Harrison et al. further observed that a combined decrease in iron and myelin content can result in $R_{2}^{*}$ decreases and unchanged susceptibility in multiple sclerosis (Harrison et al., 2016). Therefore, it seems that the $R_{2}^{*}$ and susceptibility decreases we observed in the left hippocampus of the LTLE group could be a result of a complex interplay between loss of myelinated neurons (demyelination) and brain iron redistribution/dysregulation in this region. This is supported by a recent study into iron dysregulation in TLE which found histopathological evidence for iron deposition as well as dysregulation in the hippocampi of TLE patients (Zimmer et al., 2021) resulting in more extra‐axonal iron, with iron binding and oxidative states also known to impact on susceptibility (Birkl et al., 2020).

Importantly, we also identified correlations between markers of hippocampal tissue composition and cognitive test scores. Left hippocampal $R_{2}^{*}$ was significantly correlated with letter fluency with lower $R_{2}^{*}$ being associated with lower (worse) cognitive test scores, suggesting that the degree of pathological change in hippocampal composition may directly relate to multidomain cognitive impairment. In the LTLE group, who had lower group‐wise left‐hippocampal $R_{2}^{*}$ (Figure 5), the effect of this cognitive score on $R_{2}^{*}$ was reduced. These effects are in agreement with prior imaging work that suggests the importance of a hippocampal contribution to letter fluency (Gleissner & Elger, 2001).

In the amygdala, bilateral and ipsilateral decreases in $R_{2}^{*}$ were observed in RTLE and LTLE compared to controls, respectively, suggestive of demyelination. The lack of significant susceptibility differences here may be attributed to the large within‐subject and inter‐subject variance in *χ* in this region (Supplementary Table 1). The amygdala is located very anteriorly in the mesial temporal lobe and is at the border between brain and non‐brain tissue antero‐medially. From a methodological perspective, the large variance in susceptibility in this region could be ascribed to technical factors, as BFR is known to be imperfect in the border region (Schweser et al., 2017). In TLE, the amygdala is a known structure of interest (Kullmann, 2011), with volumetric (Cendes et al., 1993) and T2 relaxometry (Kälviäinen et al., 1997) abnormalities that reflect partial sclerosis (Nakayama et al., 2017). Moreover, resection of the amygdala during temporal lobe surgery may lead to improved surgical outcomes (Schramm, 2008). All this points to similar pathological changes in the amygdala as the hippocampus, reflected by a similar $R_{2}^{*}$ decrease in these two regions.

The right thalamus had significantly higher susceptibility values in RTLE compared to controls. The positive correlation of FBTCS frequency, a clinical marker of TLE severity, with the susceptibility in the ipsilateral thalamus is concordant with these changes. $R_{2}^{*}$ measurements indicated abnormalities in the left thalamus in this RTLE group, with increased $R_{2}^{*}$ indicating increased magnetic susceptibility sources. The observed increased thalamic susceptibility and $R_{2}^{*}$ increases found here indicate that iron deposition is the most parsimonious explanation.

Left thalamus $R_{2}^{*}$ values were positively correlated with matrix reasoning, consistent with demyelination affecting cognitive performance. The elevated susceptibility and $R_{2}^{*}$ values in the ipsilateral thalamus in RTLE are more consistent with iron deposition than demyelination, indicating either disparate processes between hemispheres or multiple co‐occurring pathological processes.

Thalamic changes are widely reported in TLE, including atrophy (Caciagli et al., 2017), diffusion MRI abnormalities (Keller et al., 2013), and reorganization of functional (Allen et al., 2017; Caciagli et al., 2020; He et al., 2020) and structural connectivity patterns (Keller et al., 2014). Our findings advance our understanding of thalamic abnormalities in TLE, by indicating tissue composition changes. Further investigations would be required to explore relationships with abnormalities observed in these other imaging features, and to clarify the biological underpinnings. A complicating factor in thalamic QSM is the great intra‐thalamic variability in susceptibility, with both myelinated and unmyelinated axons, and different cellular composition of the thalamic nuclei (Li et al., 2020). Given the small sample size and relatively low SNR of our data, we consider our findings best interpreted as exploratory. Future work leveraging larger sample sizes is advocated to better establish the underlying drivers of thalamic susceptibility changes.

In the putamen, the RTLE group had a significantly higher susceptibility compared to controls in the right putamen, and a higher susceptibility compared to the LTLE group in the left putamen. There was further evidence of differences between controls and TLE patients, in that there was a significant left–right asymmetry in HCs, concordant with higher iron content in the left than right putamen (Xu et al., 2008). This asymmetry was not identified in left or right TLE patients. However, an increase in $R_{2}^{*}$ was found in the left putamen in the RTLE group compared to the LTLE group. These *χ* and $R_{2}^{*}$ findings are consistent with iron deposition in the putamen in TLE. The putamen has previously been shown to be affected in TLE patients, with smaller putamen volume bilaterally compared to HCs (Pulsipher et al., 2007).

A within‐subject asymmetry in $R_{2}^{*}$ in the GP was observed in the RTLE group, with the left GP having higher $R_{2}^{*}$ values than the right, and comparison with values from controls (Supplementary Table 1) indicates that indeed the left GP exhibits abnormally high $R_{2}^{*}$. This finding is in line with the increased $R_{2}^{*}$ in the left thalamus and left putamen in the RTLE group. Higher right GP $R_{2}^{*}$ was correlated with worse category fluency performance, which could be explained by iron deposition affecting local function. The GP was shown to be atrophic in TLE (Dabbs et al., 2012), and involved in an abnormal functional subnetwork with the putamen (He et al., 2020) and structural networks (Park et al., 2019), showing structural and functional abnormalities in line with the $R_{2}^{*}$ differences found here.

### 4.2 Correlations with clinical features

We observed significant positive correlations between left caudate $R_{2}^{*}$ and age of epilepsy onset and significant negative correlations between *χ* in the hippocampus and putamen and age of onset. Considering *χ* and $R_{2}^{*}$ correlations across these ROIs, this is consistent with reduced myelin content in those with earlier epilepsy onset.

We observed significant positive correlations between χ and $R_{2}^{*}$ and FBTCS frequency in the thalamus and GP. These are the same regions found by He et al. (2020) to have altered between‐region functional interactions in those with recent FBTCS. As such, our results provide a possible structural hypothesis of increased iron deposition underpinning those previously observed functional changes.

Cognitive impairment, as captured by neuropsychological tests, is common in TLE and encompasses multiple domains, including memory, language, information processing, and executive function (Hermann et al., 1997). Here, we found that worse neuropsychological performance correlated with changes in mean *χ* and $R_{2}^{*}$ in four ROIs on three cognitive domains, with consistent correlations for information processing (left thalamus for matrix reasoning), executive function (letter fluency in left thalamus and left hippocampus), and working memory (arithmetic in left caudate and left putamen) all concordant with demyelination.

### 4.3 Impact

To the best of our knowledge, this is the first study to use QSM in TLE patients in vivo to investigate and quantify alterations in deep grey matter structures. Therefore, comparisons will be made with literature from other neurological conditions. Several other neurological disorders, including Alzheimer's, Parkinson's, and Huntington's diseases, are associated with widespread increases in susceptibility and $R_{2}^{*}$ compared to controls that affect regions analysed in our work, including the amygdala, hippocampus, GP, thalamus, and putamen (Acosta‐Cabronero et al., 2013; Damulina et al., 2020; Thomas et al., 2020; Van Bergen et al., 2016). Given the well‐characterised hippocampal abnormalities in this population of TLE with HS, we reason that the observed decreased hippocampal susceptibility is most likely a result of focal pathology. This is supported by the intra‐subject asymmetry in susceptibility observed in the LTLE surgical subgroup alongside decreased $R_{2}^{*}$ in pathological hippocampi compared to healthy contralateral hippocampi. Similar considerations apply to the reduced $R_{2}^{*}$ in the amygdala (Nakayama et al., 2017). Increased susceptibility and $R_{2}^{*}$ observed in the putamen and thalamus in TLE suggest increased iron content in these regions. This is consistent with neurodegeneration studies where increased susceptibility in these regions was attributed to iron accumulation as part of the neurodegenerative process (Acosta‐Cabronero et al., 2013; Damulina et al., 2020; Thomas et al., 2020). There is ongoing debate as to whether epilepsy is a neurodegenerative disease (Cole, 2000; Rossini et al., 2017; Sutula et al., 2003). A recent meta‐analysis (Caciagli et al., 2017) of MRI studies over the last two decades identified progressive cortico‐subcortical grey matter loss in TLE, and recent longitudinal MRI work found progressive cortical thinning in people with focal epilepsy, including TLE, beyond that observed in healthy aging (Galovic et al., 2019). Hence, the results from our study could be interpreted as being consistent with this narrative. Further investigations are required to confirm or disprove such interpretation.

From a methodological perspective, we show that the difference in image quality between three susceptibility calculation methods – with WH‐QSM performing best here – exemplifies the impact that non‐optimized data processing can have on study results. Recent QSM challenges (Bilgic et al., 2021; Langkammer et al., 2018) have set out to ascertain which susceptibility calculation methods are most accurate and informed our choice of analysis methods. The top‐scoring FANSI method (Milovic et al., 2018), used with the reportedly most accurate TV‐based regularisation that promotes piece‐wise constant solutions, yielded higher variability in our data compared to an adapted version of FANSI with an additional WH penalty term, WH‐QSM (Milovic et al., 2019). This WH penalisation was designed to remove residual harmonic background fields (Milovic et al., 2019) and successfully did so for our data. Although there are ongoing efforts within the QSM research community to achieve consensus on the best QSM processing and susceptibility calculation methods (Schweser, 2022), this study suggests that optimisation and choice of QSM reconstruction methods for particular datasets are necessary and beneficial, particularly when performing retrospective QSM reconstruction on data acquired using parameters that were not optimised for QSM – as in this study.

## 5 LIMITATIONS

A main limitation of this work is the low SNR of the data, which was a consequence of using routinely acquired susceptibility‐weighted imaging data without QSM‐optimised acquisition parameters. We addressed this by conducting an evaluation of three QSM methods to minimise artefacts and ensure robustness in our susceptibility estimates. Furthermore, our patient sample size was relatively small, and although it was exclusively a TLE‐HS population, there was still within‐group heterogeneity in terms of age of onset and seizure characteristics, such as FBTCS frequency, which we identified as associated with these susceptibility‐based imaging measures. The consistency of our results – with matching χ and $R_{2}^{*}$ changes across participant groups, consistent correlations across different tests per cognitive domain, and findings correlating with clinical features such as FBTCS characteristics and age of onset – indicate that these results reflect genuine changes in tissue composition in TLE. The lifespan trajectories of susceptibility and $R_{2}^{*}$ with age are nonlinear (Treit et al., 2021), but a linear correction for age was selected because it provided the best fit to our data.

Although QSM and $R_{2}^{*}$ changes may reflect and suggest changes in tissue composition, the findings of this study were all obtained from non‐invasive in vivo imaging; therefore, we can only speculate about the neuropathological substrates underpinning these imaging findings. Neuropathological studies using either resected tissue or post‐mortem tissues are essential to reveal the underlying histopathological tissue changes in TLE. Note that typical anterior temporal lobe resections may be limited to the hippocampus and amygdala and thus may not help clarify histopathological changes throughout the subcortical grey matter.

## 6 CONCLUSION

In this study, we found susceptibility and $R_{2}^{*}$ abnormalities in TLE patients compared to HCs that affected the hippocampus, amygdala, thalamus, and basal ganglia. Changes observed in our TLE populations provide evidence in support of demyelination in the amygdalae and selective loss of low‐myelinated neurons combined with iron redistribution in the hippocampus, predominantly ipsilaterally, indicative of sensitivity to local HS pathology. The increased susceptibility and $R_{2}^{*}$ in the thalamus and putamen are concordant with QSM changes related to increased iron content observed in other neurological diseases and seem to reflect disease severity. Further work is required to characterise pathological hippocampal changes that precede HS in TLE and that may, in turn, lead to a decrease in hippocampal susceptibility.

## FUNDING INFORMATION

Oliver Kiersnowski was supported by EPSRC Doctoral Training Partnership (EP/R513143/1) and EPSRC‐funded UCL Centre for Doctoral Training in Intelligent, Integrated Imaging in Healthcare (i4health) (EP/S021930/1). Gavin Winston was supported by the Medical Research Council (MR/M00841X/1). Lorenzo Caciagli was supported by a scholarship from Brain Research UK (ref. 14181). Emma Biondetti was supported by the EPSRC (1489882). John Thornton, John Duncan, and Sjoerd Vos received support from the National Institute for Health Research University College London Hospitals Biomedical Research Centre. Karin Shmueli was supported by European Research Council Consolidator Grant DiSCo MRI SFN 770939.

## CONFLICT OF INTEREST

The authors report no competing interests.

## Supporting information


**SUPPLEMENTARY FIGURE 1.** Quality control: An example of a subject excluded due to motion artefacts (orange arrows). An example 3rd echo gradient‐echo magnitude image (left) and corresponding susceptibility map (right) with severe motion artefacts, resulting in exclusion of this subject from the final data set analysed in this study.
**SUPPLEMENTARY FIGURE 2**. Hippocampal volume versus hippocampal susceptibility and $R_{2}^{*}$ within groups. Scatterplots of hippocampal volume versus hippocampal susceptibility (*χ*) and $R_{2}^{*}$ within the three groups. There was a significant positive correlation between volume and $R_{2}^{*}$ in the right TLE group (p=.036). In the controls, the left hippocampi are shown in blue and the right hippocampi with black dots. In the TLE groups, hippocampi ipsilateral to hippocampal sclerosis are shown as blue crosses with contralateral hippocampi shown as black dots. TLE: temporal lobe epilepsy.
**SUPPLEMENTARY FIGURE 3**. Plots of mean susceptibility versus age for all ROIs and groups. Linear fits of susceptibility as a function of age for the three groups: healthy controls (HC), left temporal lobe epilepsy (LTLE) and right temporal lobe epilepsy (RTLE) for all ROIs, pooled across both hemispheres. The slope of the fit of the HC in the amygdala was significantly higher than the slope of the LTLE fit using analysis of covariance. No other significant differences in the slopes of fits of mean susceptibility v. age were observed.
**SUPPLEMENTARY FIGURE 4**. Plots of mean $R_{2}^{*}$ versus age for all ROIs and groups. Linear fits of $R_{2}^{*}$ as a function of age for the three groups: healthy controls (HC), left temporal lobe epilepsy (LTLE) and right temporal lobe epilepsy (RTLE) for all ROIs, pooled across both hemispheres. No fits were found to be significantly different using analysis of covariance.
**SUPPLEMENTARY TABLE 1**. Mean susceptibility (*χ*) and *R*_2_* values per group per region of interest.
**SUPPLEMENTARY TABLE 2**. Significant results of multiple linear regressions between neuropsychological test scores and susceptibility or $R_{2}^{*}$. Only those regressions that were significant after false discovery rate for multiple comparisons are shown. In these regressions, cognitive test score was a continuous variable; patient group was a binary variable: LTLE = 0 and RTLE = 1; and the outcome variable (susceptibility / $R_{2}^{*}$) was also a continuous variable.

## Data Availability

The data that support the findings of this study are available upon reasonable request from the corresponding author.
