# Supplementary material for: Quantitative susceptibility mapping identifies hippocampal and other subcortical grey matter tissue composition changes in temporal lobe epilepsy
Source: Hum Brain Mapp. 2023 Jul 26;44(15):5047–64. doi: 10.1002/hbm.26432 (PMC10502681; doi:10.1002/hbm.26432)
Supplement: Supplementary file 1 — SUPPLEMENTARY FIGURE 1. Quality control: An example of a subject excluded due to motion artefacts (orange arrows). An example 3rd echo gradient‐echo magnitude image (left) and corresponding susceptibility map (right) with severe motion artefacts, resulting in exclusion of this subject from the final data set analysed in this study. SUPPLEMENTARY FIGURE 2. Hippocampal volume versus hippocampal susceptibility and R2* within groups. Scatterplots of hippocampal volume versus hippocampal susceptibility (χ) and R2* within the three groups. There was a significant positive correlation between volume and R2* in the right TLE group (p=.036). In the controls, the left hippocampi are shown in blue and the right hippocampi with black dots. In the TLE groups, hippocampi ipsilateral to hippocampal sclerosis are shown as blue crosses with contralateral hippocampi shown as black dots. TLE: temporal lobe epilepsy. SUPPLEMENTARY FIGURE 3. Plots of mean susceptibility versus age for all ROIs and groups. Linear fits of susceptibility as a function of age for the three groups: healthy controls (HC), left temporal lobe epilepsy (LTLE) and right temporal lobe epilepsy (RTLE) for all ROIs, pooled across both hemispheres. The slope of the fit of the HC in the amygdala was significantly higher than the slope of the LTLE fit using analysis of covariance. No other significant differences in the slopes of fits of mean susceptibility v. age were observed. SUPPLEMENTARY FIGURE 4. Plots of mean R2* versus age for all ROIs and groups. Linear fits of R2* as a function of age for the three groups: healthy controls (HC), left temporal lobe epilepsy (LTLE) and right temporal lobe epilepsy (RTLE) for all ROIs, pooled across both hemispheres. No fits were found to be significantly different using analysis of covariance. SUPPLEMENTARY TABLE 1. Mean susceptibility (χ) and R 2* values per group per region of interest. SUPPLEMENTARY TABLE 2. Significant results of multiple linear regressions between neuropsy [file HBM-44-5047-s001.docx]

## **Supplementary Material**

**
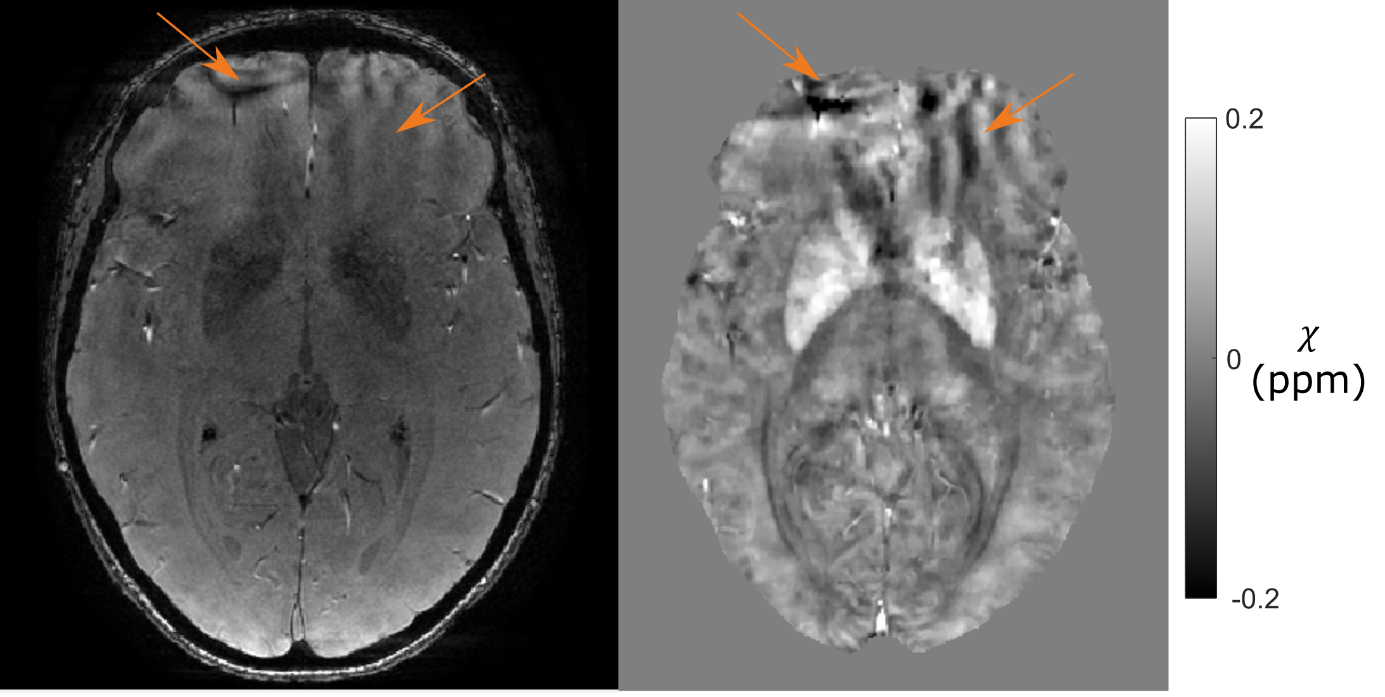
**

**Supplementary Figure 1: Quality control: An example of a subject excluded due to motion artefacts (orange arrows).** An example 3^rd^ echo gradient-echo magnitude image (left) and corresponding susceptibility map (right) with severe motion artefacts, resulting in exclusion of this subject from the final data set analysed in this study.


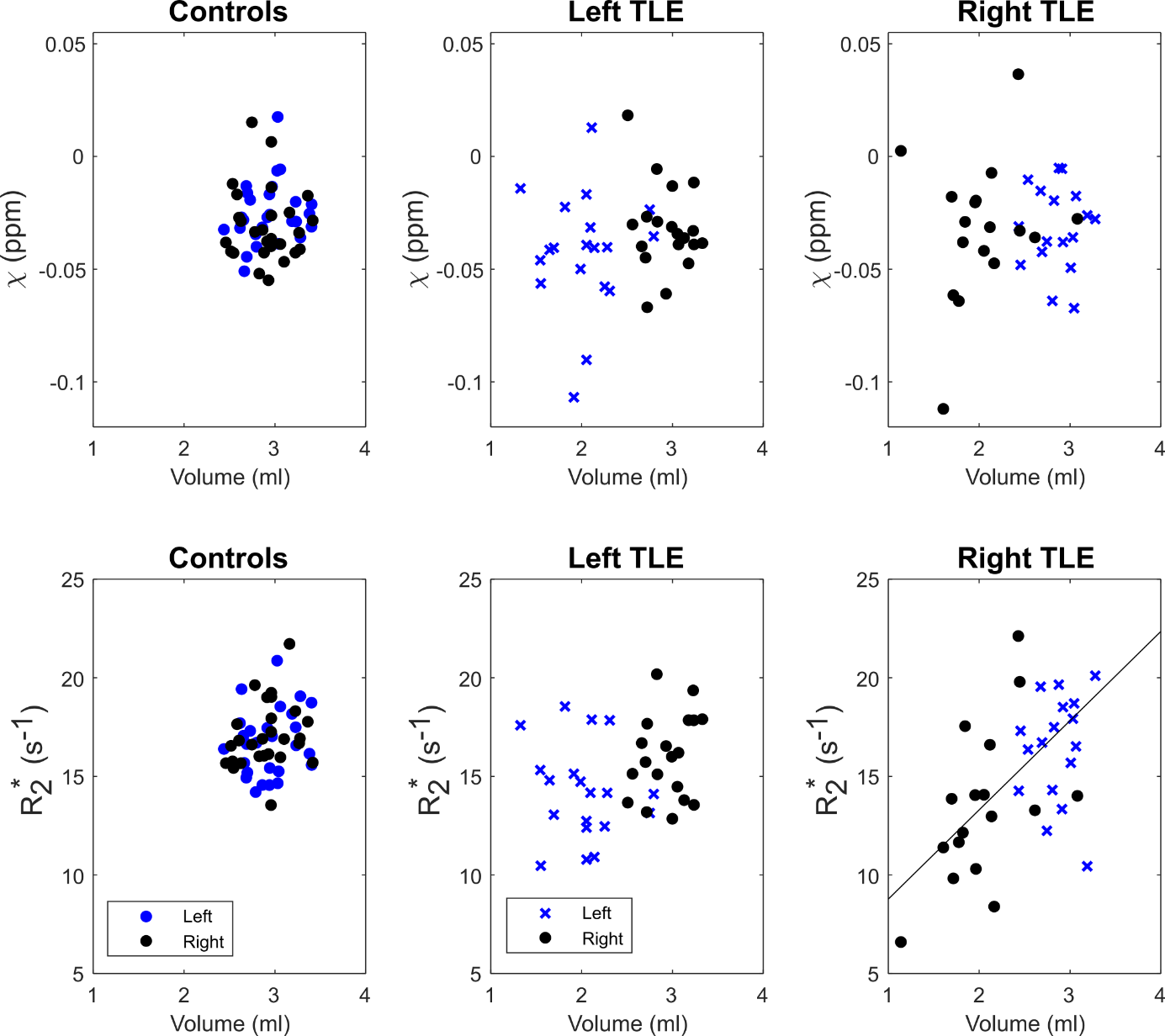


**Supplementary Figure 2: Hippocampal volume versus hippocampal susceptibility and** $\boldsymbol{R}_{\boldsymbol{2}}^{\boldsymbol{*}}$ **within groups**. Scatterplots of hippocampal volume versus hippocampal susceptibility (χ) and $R_{2}^{*}$ within the three groups. There was a significant positive correlation between volume and $R_{2}^{*}$ in the right TLE group ($P=0.036$). In the controls, the left hippocampi are shown in blue and the right hippocampi with black dots. In the TLE groups, hippocampi ipsilateral to hippocampal sclerosis are shown as blue crosses with contralateral hippocampi shown as black dots. Abbreviations: TLE: temporal lobe epilepsy.


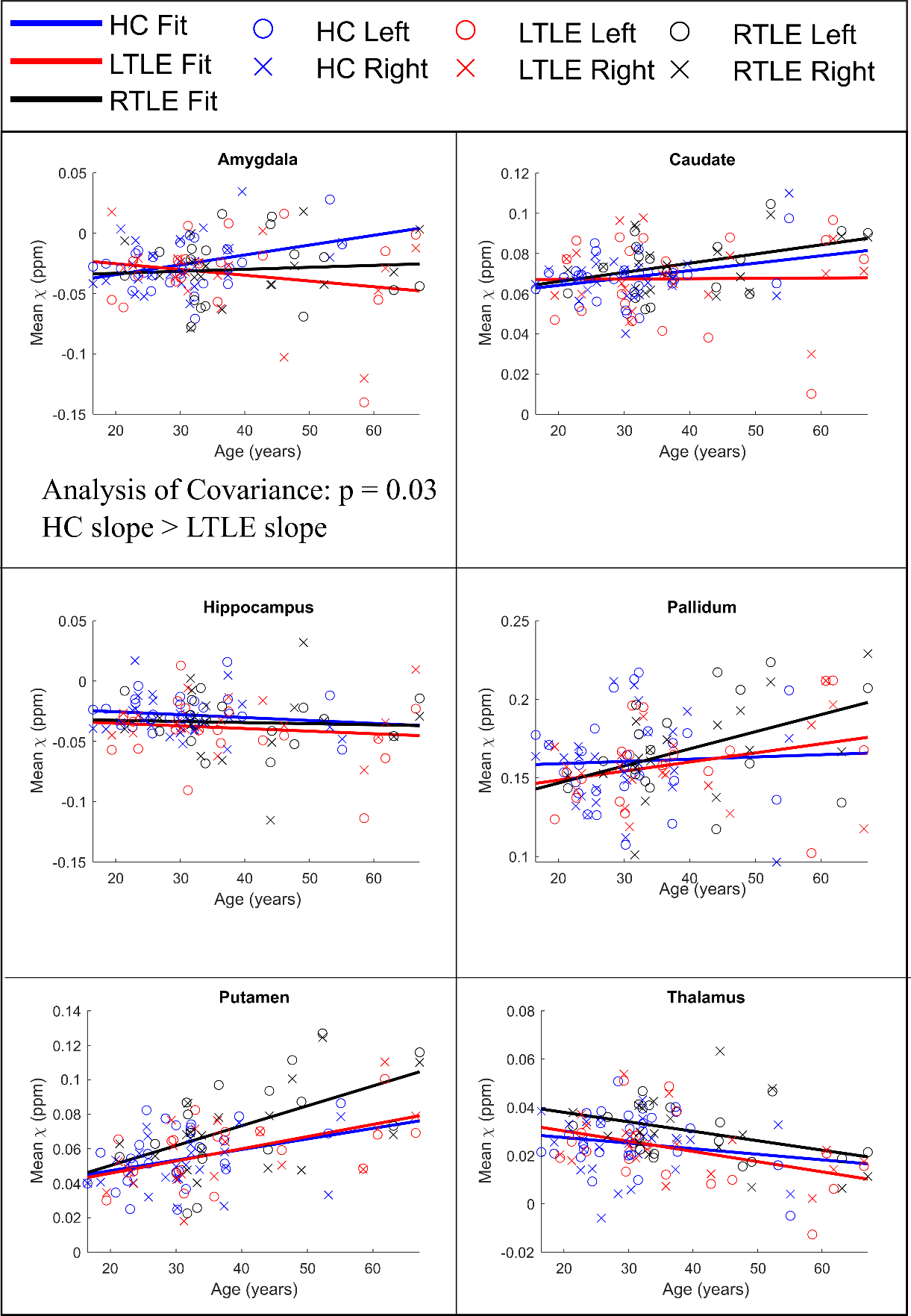


**Supplementary Figure 3: Plots of mean susceptibility versus age for all ROIs and groups**. Linear fits of susceptibility as a function of age for the three groups: healthy controls (HC), left temporal lobe epilepsy (LTLE) and right temporal lobe epilepsy (RTLE) for all ROIs, pooled across both hemispheres. The slope of the fit of the HC in the amygdala was significantly higher than the slope of the LTLE fit using analysis of covariance. No other significant differences in the slopes of fits of mean susceptibility v. age were observed.


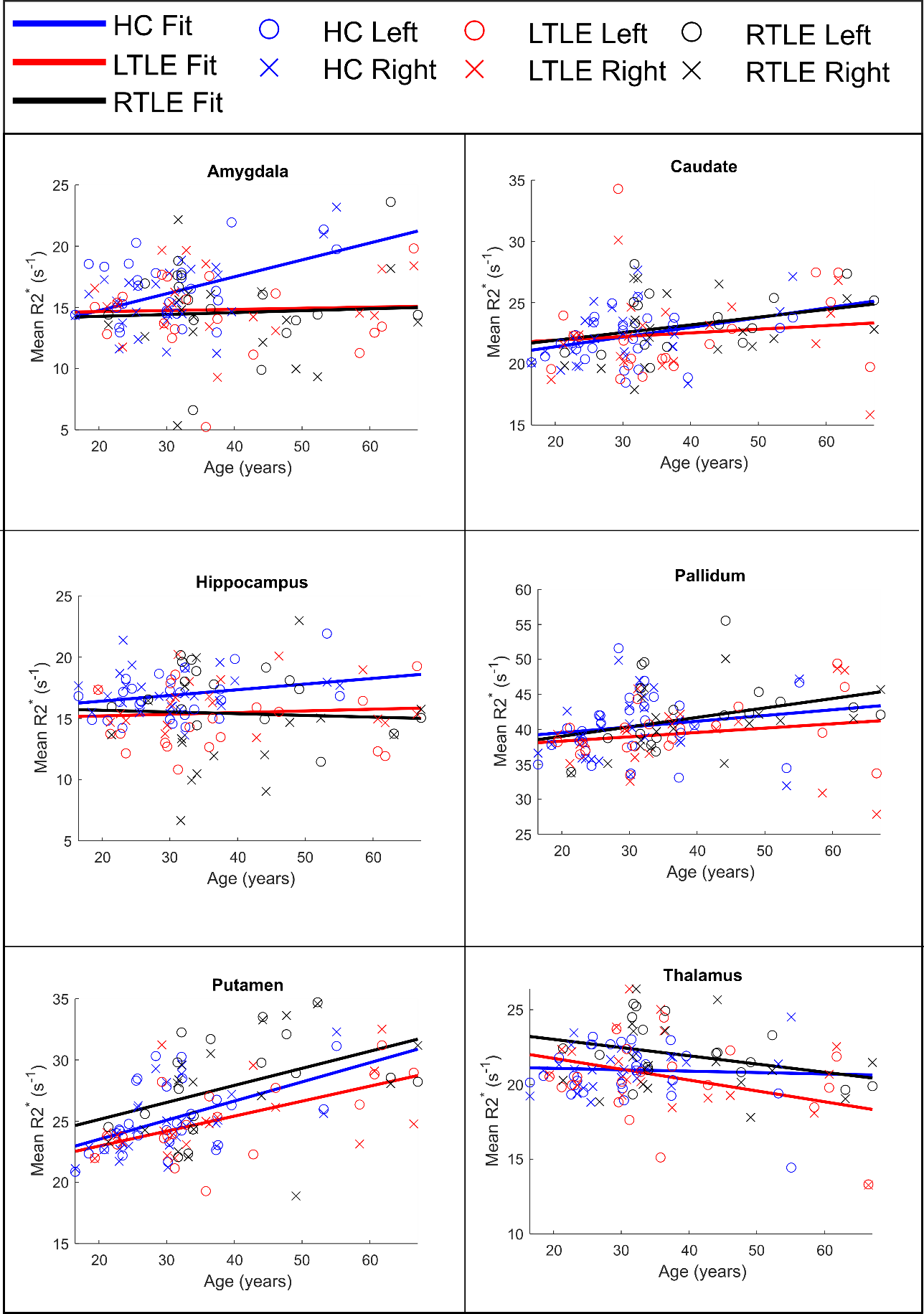


**Supplementary Figure 4: Plots of mean** $\boldsymbol{R}_{\boldsymbol{2}}^{\boldsymbol{*}}$ **versus age for all ROIs and groups**. Linear fits of $R_{2}^{*}$ as a function of age for the three groups: healthy controls (HC), left temporal lobe epilepsy (LTLE) and right temporal lobe epilepsy (RTLE) for all ROIs, pooled across both hemispheres. No fits were found to be significantly different using analysis of covariance.

**Supplementary Table 1: Mean Susceptibility (χ) and R_2_* values per group per region of interest**

| χ (10^-2^ ppm) | Healthy controls  left / right | Left TLE  left / right | Right TLE  left / right |
| --- | --- | --- | --- |
| Amygdala | -2.71 / -2.46 | -3.77 / -4.17 | -3.82 / -3.73 |
| Caudate nucleus | 6.76 / 6.84 | 6.40 / 6.56 | 7.10 / 7.24 |
| Hippocampus | -2.51 / -3.07 | -4.21 / -3.21 | -3.18 / -3.22 |
| Globus Pallidus | 16.19 / 15.91 | 15.93 / 15.63 | 16.89 / 16.55 |
| Putamen | 5.70 / 5.03 | 5.41 / 5.36 | 6.95 / 6.55 |
| Thalamus | 2.70 / 2.33 | 2.30 / 2.59 | 3.20 / 3.26 |
| $R_{2}^{*}$ (s^-1^) |  |  |  |
| Amygdala | 16.5 / 15.9 | 13.3 / 14.4 | 13.9 / 12.6 |
| Caudate nucleus | 22.0 / 22.4 | 21.9 / 21.9 | 22.8 / 22.0 |
| Hippocampus | 16.7 / 17.1 | 14.2 / 16.0 | 16.4 / 13.5 |
| Globus Pallidus | 40.6 / 40.2 | 39.1 / 38.5 | 42.0 / 39.9 |
| Putamen | 25.3 / 25.0 | 23.9 / 24.1 | 26.8 / 26.0 |
| Thalamus | 20.8 / 21.2 | 20.1 / 20.9 | 22.4 / 21.6 |

Abbreviations: TLE: temporal lobe epilepsy

**Supplementary Table 2: Significant results of multiple linear regressions between neuropsychological test scores and susceptibility or** $\boldsymbol{R}_{\boldsymbol{2}}^{\boldsymbol{*}}$**.** Only those regressions that were significant after false discovery rate for multiple comparisons are shown. In these regressions, cognitive test score was a continuous variable; patient group was a binary variable: LTLE=0 and RTLE=1; and the outcome variable (susceptibility / $R_{2}^{*}$) was also a continuous variable.

|  | **Left caudate susceptibility and arithmetic**  Whole model: p=0.0032, adjusted R^2^: 0.53, n = 18 | |
| --- | --- | --- |
|  | Beta | p-value |
| Intercept | 0.032 | 0.0045 |
| Patient group | 0.11 | 0.0023 |
| Cognitive score | 3.97×10^-3^ | 0.0018 |
| Cognitive × group interaction | -0.011 | 0.0026 |
|  |  |  |
|  | **Left putamen susceptibility and arithmetic**  Whole model: p<0.001, adjusted R^2^: 0.68, n = 18 | |
|  | Beta | p-value |
| Intercept | 0.026 | 0.031 |
| Patient group | 0.19 | 5.05×10^-3^ |
| Cognitive score | 3.21×10^-3^ | 0.019 |
| Cognitive × group interaction | -0.019 | 1.22×10^-4^ |
|  |  |  |
|  | **Left hippocampal** $\boldsymbol{R}_{\boldsymbol{2}}^{\boldsymbol{*}}$ **and letter fluency**  Whole model: p=0.0022, adjusted R^2^: 0.34, n = 32 | |
|  | Beta | p-value |
| Intercept | 12.63 | 2.42×10^-12^ |
| Patient group | -0.36 | 0.85 |
| Cognitive score | 0.091 | 0.23 |
| Cognitive × group interaction | 0.20 | 0.13 |
|  |  |  |
|  | **Left thalamic** $\boldsymbol{R}_{\boldsymbol{2}}^{\boldsymbol{*}}$ **and letter fluency**  Whole model: p=0.0056, adjusted R^2^: 0.29, n = 32 | |
|  | Beta | p-value |
| Intercept | 19.08 | 7.96×10^-19^ |
| Patient group | 0.89 | 0.57 |
| Cognitive score | 0.092 | 0.15 |
| Cognitive × group interaction | 0.76 | 0.48 |
|  |  |  |
|  | **Left thalamic** $\boldsymbol{R}_{\boldsymbol{2}}^{\boldsymbol{*}}$ **and matrix reasoning**  Whole model: p=0.0029, adjusted R^2^: 0.33, n = 31 | |
|  | Beta | p-value |
| Intercept | 17.46 | 1.83×10^-12^ |
| Patient group | 2.32 | 0.25 |
| Cognitive score | 0.38 | 0.041 |
| Cognitive × group interaction | -0.053 | 0.82 |

Abbreviations: LTLE, left temporal lobe epilepsy; RTLE, right temporal lobe epilepsy
